# Transcriptional profiling reveals developmental relationship and distinct biological functions of CD16+ and CD16- monocyte subsets

**DOI:** 10.1186/1471-2164-10-403

**Published:** 2009-08-27

**Authors:** Petronela Ancuta, Kuang-Yu Liu, Vikas Misra, Vanessa Sue Wacleche, Annie Gosselin, Xiaobo Zhou, Dana Gabuzda

**Affiliations:** 1 CRCHUM, Université de Montréal, INSERM Unit 743, Montréal, Québec, Canada; 2 Department of Anesthesiology, Perioperative and Pain Medicine, Brigham and Women's Hospital, Harvard Medical School, Boston, MA, USA; 3 Department of Cancer Immunology and AIDS, Dana-Farber Cancer Institute, Harvard Medical School, Boston, MA, USA; 4 Bioinformatics Core and Department of Radiology, The Methodist Hospital Research Institute, Weill Cornell Medical College, Houston, TX 77030, USA

## Abstract

**Background:**

Human peripheral blood monocytes (Mo) consist of subsets distinguished by expression of CD16 (FCγRIII) and chemokine receptors. Classical CD16^- ^Mo express CCR2 and migrate in response to CCL2, while a minor CD16^+ ^Mo subset expresses CD16 and CX3CR1 and migrates into tissues expressing CX3CL1. CD16^+ ^Mo produce pro-inflammatory cytokines and are expanded in certain inflammatory conditions including sepsis and HIV infection.

**Results:**

To gain insight into the developmental relationship and functions of CD16^+ ^and CD16^- ^Mo, we examined transcriptional profiles of these Mo subsets in peripheral blood from healthy individuals. Of 16,328 expressed genes, 2,759 genes were differentially expressed and 228 and 250 were >2-fold upregulated and downregulated, respectively, in CD16^+ ^compared to CD16^- ^Mo. CD16^+ ^Mo were distinguished by upregulation of transcripts for dendritic cell (DC) (SIGLEC10, CD43, RARA) and macrophage (MΦ) (CSF1R/CD115, MafB, CD97, C3aR) markers together with transcripts relevant for DC-T cell interaction (CXCL16, ICAM-2, LFA-1), cell activation (LTB, TNFRSF8, LST1, IFITM1-3, HMOX1, SOD-1, WARS, MGLL), and negative regulation of the cell cycle (CDKN1C, MTSS1), whereas CD16^- ^Mo were distinguished by upregulation of transcripts for myeloid (CD14, MNDA, TREM1, CD1d, C1qR/CD93) and granulocyte markers (FPR1, GCSFR/CD114, S100A8-9/12). Differential expression of CSF1R, CSF3R, C1QR1, C3AR1, CD1d, CD43, CXCL16, and CX3CR1 was confirmed by flow cytometry. Furthermore, increased expression of RARA and KLF2 transcripts in CD16^+ ^Mo coincided with absence of cell surface cutaneous lymphocyte associated antigen (CLA) expression, indicating potential imprinting for non-skin homing.

**Conclusion:**

These results suggest that CD16^+ ^and CD16^- ^Mo originate from a common myeloid precursor, with CD16^+ ^Mo having a more MΦ – and DC-like transcription program suggesting a more advanced stage of differentiation. Distinct transcriptional programs, together with their recruitment into tissues *via *different mechanisms, also suggest that CD16^+ ^and CD16^- ^Mo give rise to functionally distinct DC and MΦ *in vivo*.

## Background

Peripheral blood monocytes (Mo) originate from hematopoietic progenitor cells in bone marrow and play important roles in innate and adaptive immunity due to their ability to differentiate into macrophages (MΦ) and dendritic cells (DC) [1-7]. The heterogeneity and plasticity of MΦ and DC result from their differentiation in specific tissue microenvironments [8-10]. The expression of CD16 (FcγRIII) distinguishes two Mo subsets in peripheral blood of healthy individuals: a major CD16^- ^subset (80–95%) and a minor CD16^+ ^subset (5–15%) [11]. Compared to classical CD16^- ^Mo, CD16^+ ^Mo exhibit a more MΦ-like morphology, produce higher levels of TNF and IL-1 [12,13], have higher antigen presenting potential [14-16], and differentiate into DC upon transendothelial migration *in vitro *[17]. CD16^+ ^Mo express CX3CR1 and migrate in response to CX3CL1 [18,19], a membrane-bound chemokine expressed on inflamed endothelial cells, while CD16^- ^Mo express CD62L and CCR2 and migrate in response to CCL2 [18,20], which mediates Mo migration from bone marrow and recruitment to inflammatory sites [2,21]. CD16^+ ^Mo produce IL-6, CCL2, and matrix metalloproteinase-9 upon interaction with CX3CL1-expressing endothelial cells [22] and activate resting T-cells for HIV infection by producing CCR3 and CCR4 ligands [23]. Together, these findings suggest that CD16^+ ^and CD16^- ^Mo are recruited into different anatomic sites under constitutive or inflammatory conditions, and play distinct functional roles in immunity and disease pathogenesis.

A dramatic increase in circulating CD16^- ^Mo has been reported in inflammatory pathologies such as sepsis, HIV infection, tuberculosis, and asthma [11,24,25]. Studies of patients infected with *Mycobacterium leprae *demonstrated that CD16^+ ^and CD16^- ^Mo differentiate into DC-SIGN^+ ^MΦ and CD1b^+^DC-SIGN^- ^DC, respectively, and the presence of CD1b^+^DC-SIGN^- ^DC in *M. leprae *lesions was associated with healing [26]. An increased frequency of CD16^+ ^Mo was associated with non-healing *Leishmania chagasi *lesions [27]. Thus, CD16^+ ^and CD16^- ^Mo differentiation into MΦ or DC subpopulations with distinct phenotypes influences host defenses in infectious disease. Consequently, there is interest in developing therapeutic strategies that target specific Mo subpopulations [6,8,28,29].

Mo heterogeneity is conserved across mammalian species [7,8,19,30]. In mice, Gr1^+^CX3CR1^low ^Mo (homolog of human CD16^- ^Mo) are recruited into the peritoneal cavity or draining lymph nodes under inflammatory conditions by mechanisms dependent on CCR2 and CD62L, and subsequently differentiate into DC [19,31-33]. In contrast, Gr1^-^CX3CR1^high ^(homolog of human CD16^+ ^Mo) are constitutively recruited into peripheral tissues including spleen, gut, lungs, and brain [19]. Gr1^-^CX3CR1^high ^patrol vascular endothelium by mechanisms involving LFA-1 and CX3CR1, and are rapidly recruited into inflamed tissues where they differentiate into MΦ expressing the transcription factors cMaf and MafB and transiently producing TNF-α [33]. Studies in CX3CR1/ApoE double knockout mice suggest that CX3CR1^high ^Mo play a critical role in development of atherosclerotic lesions [34,35]. CX3CR1^+ ^Mo may be precursors for lamina propria DC, which depend on CX3CR1 to form transepithelial dendrites, enabling direct sampling of luminal antigens [36]. Furthermore, adoptive transfer studies in rats demonstrated that CCR2^low^CX3CR1^high ^Mo are constitutively recruited into the gut where they give rise to intestinal lymph DC [37]. Studies on the origin of myeloid pulmonary DC demonstrated that Ly-6C^high^CCR2^high^CX3CR1^low ^Mo differentiate into CD103^+ ^DC [38,39]), whereas Ly-6C^low^CCR2^low^CX3CR1^high ^Mo give rise to CD11b^high ^DC [39,40]. Thus, CX3CR1^high ^and CX3CR1^low ^Mo subsets play distinct functional roles under constitutive and inflammatory conditions.

The developmental relationship between Mo subsets is poorly understood. In mice, Mo recently emigrating from bone marrow exhibit a Ly-6C^high ^phenotype and gradually downregulate Ly-6C [41]. Mo acquire CD16 expression upon exposure to M-CSF [42], TGF-β [17,43], or IL-10 [44], and upregulate CX3CR1 expression upon CCL2 stimulation via CCR2 [45]. Mo differentiation is associated with decreased CCR2 expression and increased CCL2 production [46]. Engrafted Gr1^high^CX3CR1^low ^Mo in peripheral blood traffic to the bone marrow, differentiate into Gr1^low^CX3CR1^high ^Mo, and contribute to mucosal, but not splenic, generation of DC [5]. These findings suggest that human CD16^+ ^Mo and mouse Ly-6C^low^CCR2^low^CX3CR1^high ^Mo differentiate from CD16^- ^Mo and Gr1^-^Ly-6C^high^CCR2^high^CX3CR1^low ^Mo, respectively.

Here, we investigate the developmental and functional relationship between CD16^+ ^and CD16^- ^Mo subsets. Whole genome transcriptome analysis suggests that these Mo subsets originate from a common myeloid precursor, with CD16^+ ^Mo being at a more advanced stage of differentiation and having a more MΦ – and DC-like transcription program. Upregulation of the transcription factors RARA and KLF2 in CD16^+ ^Mo coincided with the absence of cutaneous lymphocyte associated antigen (CLA) expression, indicating potential imprinting for non-skin homing in CD16^+ ^Mo. These results define distinct transcriptional profiles of CD16^- ^and CD16^+ ^Mo subsets suggesting different stages of myeloid differentiation, new markers to distinguish these Mo subpopulations, and unique roles in immune responses and inflammatory diseases.

## Methods

### Antibodies

Fluorochrome-conjugated Abs used for FACS analysis were CD14, CD16, CD19, CD16b, CD66b, CD56, and CD3 (Beckman Coulter); M-DC8 and CD1c (Miltenyi), HLA-DR, CD114, C3aR, CD1d and CD43 (BD Pharmingen), CD115 (R&D Systems), CD93/C1qR1 (Chemicon International), and CXCL16 (R&D Systems). Matched isotype controls were from the same source as the Abs.

### Flow cytometry analysis

Blood from healthy individuals was collected with informed consent and IRB approval from Dana-Farber Cancer Institute. PBMC isolated from peripheral blood by Ficoll-Paque gradient density centrifugation were stained with fluorochrome-conjugated Abs and analyzed by multi-color flow cytometry (BD FACSCalibur or LSRII).

### Monocyte sorting

Monocytes (Mo) were isolated by negative selection using magnetic immunobeads (Monocyte Isolation Kit II, Miltenyi) as described [18,47]. The purity of sorted Mo was >98%, as determined by FACS analysis indicating the expression of CD14 and HLA-DR (monocyte markers) and absence of CD1c (DC marker), CD56 (NK cell marker), CD19 (B cell marker), CD3 (T cell marker), and CD16b and CD66b (neutrophil markers) expression. CD16^+ ^and CD16^- ^Mo fractions were further isolated using CD16 magnetic immunobeads (Miltenyi) with >85% and >95% purity for CD16^+ ^and CD16^- ^Mo fractions, respectively, as determined by FACS analysis after staining with CD16 Abs [23]. Mo fractions isolated under RNase free conditions were stored in Trizol at -80°C for subsequent RNA extraction.

### RNA isolation and microarray analysis

Total RNA from Mo pellets was isolated by Trizol extraction and purified using RNeasy columns (Qiagen). The quality of RNA was assessed by visualization of intact bands corresponding to 18S and 28S rRNA on formaldehyde agarose gels. Total RNA (10 μg) from matched CD16^+ ^and CD16^- ^Mo samples isolated from 4 different healthy donors was quality tested using an Agilent 2100 Bioanalyzer chip, reverse transcribed, and hybridized on the GeneChip^® ^Human Genome U133 Plus 2.0 Array (Affymetrix), which includes 54,000 probe sets on a single array (*i.e.*, 47,000 transcripts and variants, including 38,500 well-characterized human genes). Primary data analysis performed using GeneSpring software (Biopolymer core facility, Harvard Medical School) generated Excel spreadsheets with relative gene expression values for the 4 matched CD16^+ ^and CD16^- ^Mo subsets.

### Microarray data analysis

A total of 16,328 probe sets were detected in these 8 samples (present calls, defined as probe sets detected in at least 3 samples). Normalization was performed as described [48] to account for variation between microarrays. Missing value estimation was performed using a modified KNN algorithm [49,50]. T-test was used to identify probe sets differentially expressed in CD16^+ ^and CD16^- ^Mo (p < 0.05). These genes were sorted according to their t-statistics and fold change ratios, which were calculated by computing the mean expression in CD16^+ ^and CD16^- ^Mo. Clustering analysis using fuzzy-c-means [50,51] was performed based on the genes selected by F-test (n = 2,759 probe sets). False discovery rates (FDR) [52] were estimated using dChip software (build date: Jan 27 2009) [53] by performing 100 random permutations using all 8 samples with p-values < 0.05, expression ratio cut-off = 2.0-fold, and present call cut-off of 20%, yielding a median FDR of 0.07. Expression ratios for differentially expressed probe sets were calculated in CD16^+ ^*versus *CD16^- ^Mo (cut-off 2-fold; p < 0.05). Heat maps for biological function categories were generated by dChip software using signal values from each of the 8 samples for genes that were > 2-fold upregulated or downregulated in CD16^+ ^Mo compared to CD16^- ^Mo. The entire microarray dataset and technical information requested by Minimum Information about a Microarray Experiment (MIAME) are available at the Gene Expression Omnibus (GEO) database under accession number GSE16836 (*Transcriptional profiling of CD16^+ ^and CD16^- ^peripheral blood monocytes from healthy individuals*) .

### Gene set enrichment analysis (GSEA)

Gene set enrichment analysis (GSEA) and Molecular Signature DataBase (MSigDB)  were used to identify differentially expressed gene sets [54]. GSEA is a computational method that determines whether an a priori defined set of genes shows statistically significant concordant differences between two biological states (e.g. phenotypes). MSigDB contains more than 3000 gene sets for use with GSEA. An enrichment score (ES) that represents the difference between the observed and expected rankings from phenotype correlation was calculated for every gene set. A nominal p-value for the specific ES was then estimated from an empirical permutation-based null distribution that preserves the complex correlation structure of the gene expression data. Multiple testing was corrected via the FDR, with FDR less than 10% considered statistically significant.

### Quantitative Real time RT-PCR

One step SYBR Green real time RT-PCR (Qiagen) was carried out in an iCycler BioRad EN270 PCR machine according to manufacturer's recommendations. Absolute quantification of target gene expression was performed using a 10-fold serial dilution of purified PCR products as described [55]. Briefly, 25–50 ng total RNA was reverse transcribed in 25 μl 1× SYBR Green mix (Qiagen) containing 0.5 μM primers, and 10 nM fluorescein calibration dye (Bio-Rad). Agarose gel electrophoresis was used to determine the size of amplification products (100–200 bp) and allowed cDNA purification (QIAquick Gel Extraction Kit; Qiagen) for standard curve preparation (i.e., 200, 20, 2, 0.2, and 0.02 fg cDNA). Primers spanning one or multiple exons were purchased from Qiagen (i.e., SIGLEC10, MafB, C1QR, C3AR1, CDKN1C, CSF1R, CSF3R, FcγRIII, TNFRSF8, ICAM-2 QuantiTect primer sets). Samples without template and reverse transcriptase were used as negative controls. The concentration of each gene was normalized to the 28S ribosomal RNA (RRN28S) internal control [55]. Each RT-PCR reaction was performed in triplicate.

## Results

### Distinct gene expression profiles in CD16^+ ^and CD16^- ^monocytes

**Figure 1:**
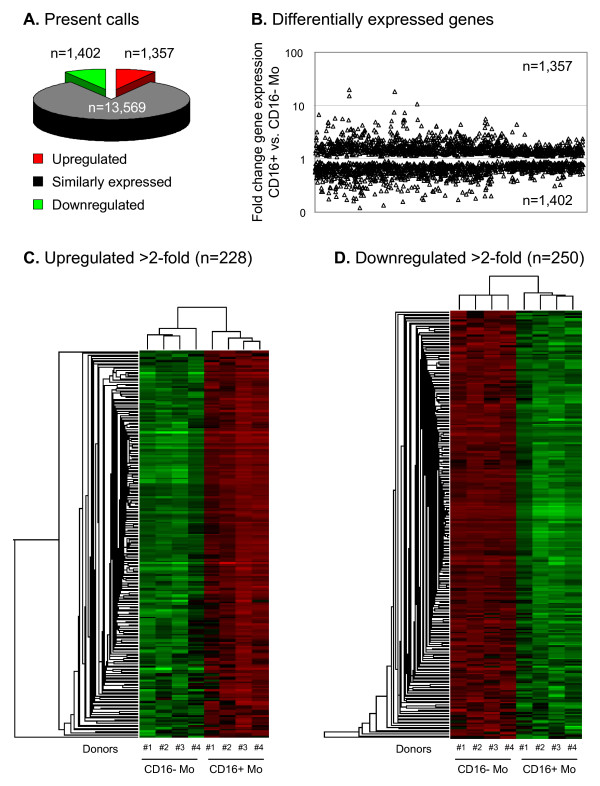
**Genome wide transcriptome analysis identifies new markers for CD16^+ ^and CD16^- ^monocyte (Mo) subsets**. **(A) **Total RNA from matched CD16^+ ^and CD16^- ^Mo samples isolated from 4 different healthy donors were reverse transcribed and hybridized on GeneChip^® ^Human Genome U133 Plus 2.0 Arrays (Affymetrix). Statistical analyses using one way ANOVA was performed to identify differentially expressed genes (p < 0.05). Graph depicts the number of probe sets shared or differentially expressed between CD16^+ ^and CD16^- ^Mo. **(B) **Graph depicts the fold change expression of probe sets differentially expressed in CD16^+ ^*versus *CD16^- ^Mo. **(C-D) **Hierarchical clustering analysis based on c-fuzzy means separated the 8 samples in 2 groups that perfectly matched CD16^+ ^and CD16^- ^Mo; heat maps were generated using differentially expressed genes (>2-fold). Red and green signify increased and decreased gene expression, respectively.

To define transcriptional profiles of monocyte subsets *in vivo*, we performed genome wide transcriptome analysis of matched CD16^+ ^and CD16^- ^Mo subsets in peripheral blood of four healthy individuals. We identified 2,759 probe sets that were differentially expressed and 13,569 genes that were similarly expressed in these Mo subsets (Figure 1A) (GEO database accession number GSE16836, ). Clustering analysis separated the 8 samples into 2 groups that perfectly matched CD16^+ ^and CD16^- ^Mo, with 1,402 genes downregulated and 1,357 genes upregulated in CD16^+ ^compared to CD16^- ^Mo (Figure 1B). Calculation of expression ratios for 2,759 differentially expressed probe sets showed that 250 probe sets were downregulated (corresponding to 166 genes and 23 unknown transcribed sequences) and 228 probe sets were upregulated (corresponding to 153 genes and 19 unknown transcribed sequences) in CD16^+ ^compared to CD16^- ^Mo (cut-off 2-fold; p < 0.05) (Figure 1C–D, Additional files 1, 2). These 2-fold lists of differentially expressed genes included known markers for CD16^+ ^(i.e., FCGR3A/CD16, CX3CR1, ITGAL/LFA-1, and CD31/PECAM1) and CD16^- ^Mo (i.e., CD14, CCR2, SELL/CD62L, FCGR1/CD64) [18-20] (Additional files 1, 2), providing initial validation of microarray results. Signature transcripts for other blood cell lineages were absent in both CD16^+ ^and CD16^- ^Mo (i.e., CD3 and CD8 for T cells, CD56 for NK cells, CD19 for B cells, and DC-SIGN and CD1c for DC), consistent with results obtained by flow cytometry demonstrating the purity of sorted Mo (>98%) and absence of DC (i.e., CD1c), NK cell (i.e., CD56), B cell (i.e., CD19), T cell (i.e., CD3), neutrophil (i.e., CD16b and CD66b) markers on CD16^+ ^and CD16^- ^Mo. The difference in relative expression of some probe sets for donor #1 probably reflects normal donor-to-donor variability, since post-sort cell viability, RNA quality, and MicroArray Quality Controls were similar for the four donors. A more stringent analysis was performed where in addition to a cut-off >2-fold and p-value < 0.05, probe sets with expression levels >3-fold higher than background were selected; by this approach, we identified 132 downregulated and 183 upregulated probe sets in CD16^+ ^compared to CD16^- ^Mo (data not shown). These genes were further selected for those with the highest levels of expression (>10,000 AU (arbitrary units), cut-off >2-fold; p-value < 0.05) and two lists of top genes were generated, with 30 and 31 transcripts upregulated in CD16^+ ^and CD16^- ^Mo, respectively (Tables 1 and 2). Other genes were differentially expressed with a difference <2-fold. In CD16^+ ^compared to CD16^- ^Mo, downregulated markers included the early myeloid markers CD13 (2,971 ± 1,753 *versus *5,656 ± 2,392; ratio 0.53, p-value < 0.05) and CD33 (1,860 ± 703 *versus *3,478 ± 686; ratio 0.52, p-value < 0.05). Thus, despite a high level of transcriptional similarity (approximately 83%), a subset of probe sets were significantly downregulated (n = 250) or upregulated (n = 228) in CD16^+ ^compared to CD16^- ^Mo, suggesting that these Mo subsets represent different stages of myeloid differentiation and have distinct biological functions *in vivo*.

**Table 1:** Top genes upregulated in CD16^+ ^compared to CD16^- ^Mo

			**CD16+ Mo AU**		
				
**Gene Symbol**	**CD16+/CD16- Ratio**	**p-value**	**Mean**	**SD**	**GeneTitle**
FCGR3A	20,1	0,000	36975	6660	Fc fragment of IgG, low affinity IIIa, receptor for (CD16)
CDKN1C	18,4	0,000	23838	5132	cyclin-dependent kinase inhibitor 1C (p57, Kip2)
MTSS1	5,7	0,000	12285	2678	metastasis suppressor 1
SIGLEC10	4,8	0,000	10657	3068	sialic acid binding Ig-like lectin 10
IFITM1	4,5	0,026	13823	10613	interferon induced transmembrane protein 1 (9–27)
HMOX1	3,5	0,000	16791	4339	heme oxygenase (decycling) 1
TAGLN	3,2	0,000	11950	3480	Transgelin
TCF7L2	3,0	0,000	16772	4608	transcription factor 7-like 2 (T-cell specific, HMG-box)
MS4A7	2,8	0,000	22645	6376	membrane-spanning 4-domains, subfamily A, member 7
CSF1R	2,8	0,000	22838	6537	colony stimulating factor 1 receptor
NAP1L1	2,8	0,000	34370	5546	nucleosome assembly protein 1-like 1
IFITM2	2,5	0,000	58835	17536	interferon induced transmembrane protein 2 (1-8D)
SOD1	2,5	0,000	11300	2491	superoxide dismutase 1
IFITM3	2,5	0,022	40793	15947	interferon induced transmembrane protein 3 (1-8U)
LST1	2,5	0,000	42904	13661	leukocyte specific transcript 1
CX3CR1	2,4	0,041	25882	11806	chemokine (C-X3-C motif) receptor 1
LILRB1	2,4	0,000	15534	4951	leukocyte immunoglobulin-like receptor, subfamily B, member 1
PSCDBP	2,3	0,000	12459	2539	pleckstrin homology, Sec7 and coiled-coil domains, binding protein
ITGAL	2,3	0,000	13269	3384	integrin, alpha L (antigen CD11A)
C6orf187	2,3	0,022	14752	5263	chromosome 6 open reading frame 187
KLF2	2,3	0,000	23783	7614	Kruppel-like factor 2 (lung)
WARS	2,3	0,000	18466	5567	tryptophanyl-tRNA synthetase
MAFB	2,3	0,000	16707	5853	v-maf musculoaponeurotic fibrosarcoma oncogene homolog B
GCH1	2,2	0,022	10956	3586	GTP cyclohydrolase 1 (dopa-responsive dystonia)
CD97	2,2	0,000	10566	3203	CD97 antigen
CTSC	2,2	0,000	10079	2619	cathepsin C
PIK3AP1	2,1	0,000	16825	4941	phosphoinositide-3-kinase adaptor protein 1
MAIL	2,1	0,000	15859	4883	molecule possessing ankyrin repeats induced by lipopolysaccharide
LYN	2,1	0,000	19388	3520	v-yes-1 Yamaguchi sarcoma viral related oncogene homolog
BCL2A1	2,1	0,022	11110	5056	BCL2-related protein A1
PECAM1	2,0	0,000	22679	7106	platelet/endothelial cell adhesion molecule (CD31 antigen)

Shown are differentially expressed genes (>2-fold higher in CD16^+ ^compared to CD16^- ^Mo) with the highest expression levels (>10,000 AU). AU, arbitrary units.

**Table 2:** Top genes upregulated in CD16^- ^compared to CD16^+ ^Mo

			**CD16- Mo AU**		
				
**Gene Symbol**	**CD16+/CD16- Ratio**	**p-value**	**Mean**	**SD**	**GeneTitle**
S100A12	0,1	0,000	32609	4915	S100 calcium binding protein A12 (calgranulin C)
CSPG2	0,2	0,000	44600	1191	chondroitin sulfate proteoglycan 2 (versican)
CD14	0,2	0,000	29424	2503	CD14 antigen
CD36	0,2	0,000	10283	2743	CD36 antigen (collagen type I receptor, thrombospondin receptor)
CD99	0,2	0,000	11219	925	CD99 antigen
DREV1	0,3	0,000	16902	2260	DORA reverse strand protein 1
CSF3R	0,3	0,000	15383	2884	colony stimulating factor 3 receptor (granulocyte)
FLJ22662	0,3	0,000	24777	3886	hypothetical protein FLJ22662
MS4A6A	0,3	0,000	17295	2171	membrane-spanning 4-domains, subfamily A, member 6A
ITGAM	0,3	0,000	10344	2182	integrin, alpha M (complement component receptor 3)
SELL	0,3	0,038	18878	6604	selectin L (lymphocyte adhesion molecule 1)
CRTAP	0,4	0,000	13026	934	cartilage associated protein
S100A9	0,4	0,000	91116	15677	S100 calcium binding protein A9 (calgranulin B)
GPX1	0,4	0,000	35555	1707	glutathione peroxidase 1
PLP2	0,4	0,000	10014	1672	proteolipid protein 2 (colonic epithelium-enriched)
S100A8	0,4	0,000	114633	17191	S100 calcium binding protein A8 (calgranulin A)
PPBP	0,4	0,025	10279	3787	pro-platelet basic protein (chemokine (C-X-C motif) ligand 7)
FPR1	0,4	0,000	23743	4367	formyl peptide receptor 1
EGFL5	0,4	0,000	10378	1126	EGF-like-domain, multiple 5
MNDA	0,4	0,000	26939	7235	myeloid cell nuclear differentiation antigen
KCTD12	0,4	0,000	21488	1640	potassium channel tetramerisation domain containing 12
DKFZp434L142	0,4	0,000	10904	1594	hypothetical protein DKFZp434L142
GRN	0,5	0,000	22341	2163	granulin
LYZ	0,5	0,038	86499	26260	lysozyme (renal amyloidosis)
APLP2	0,5	0,000	23441	2191	amyloid beta (A4) precursor-like protein 2
ALDH2	0,5	0,000	14898	1814	aldehyde dehydrogenase 2 family (mitochondrial)
HIF1A	0,5	0,000	14221	3447	hypoxia-inducible factor 1, alpha subunit
TALDO1	0,5	0,000	21276	2781	transaldolase 1
IRF2BP2	0,5	0,000	21329	4404	interferon regulatory factor 2 binding protein 2
EVI2A	0,5	0,000	10200	1342	ecotropic viral integration site 2A
AMICA	0,5	0,000	10160	945	adhesion molecule AMICA
DPYD	0,5	0,000	10049	1957	dihydropyrimidine dehydrogenase

Shown are differentially expressed genes (>2-fold higher in CD16^- ^compared to CD16^+ ^Mo) with the highest expression levels (>10,000 AU). AU, arbitrary units.

### Validation of microarray results and identification of new surface markers that distinguish CD16^+ ^and CD16^- ^monocytes

**Figure 2:**
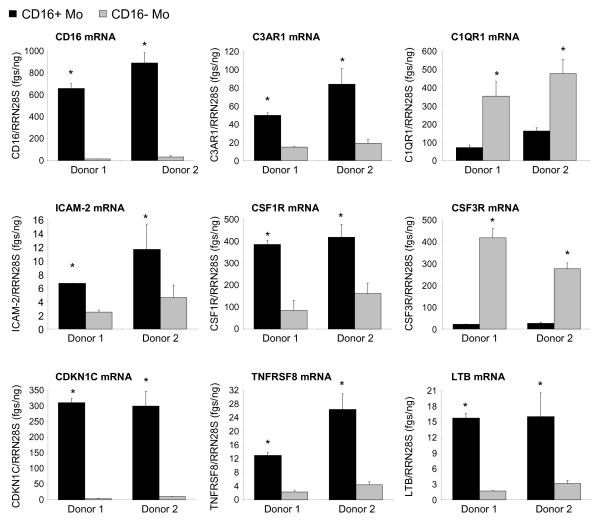
**Real-time RT-PCR validation of microarray results**. The expression of CD16, C3AR1, C1QR1, ICAM-2, CSFR1, CSF3R, CDKN1C, TNFRSF8, and LTB mRNA was quantified by SYBR Green real time RT-PCR in CD16^+ ^and CD16^- ^Mo. The concentration of each gene was normalized to the 28S rRNA internal control and expressed as fgs RNA of a target gene per 1 ng rRNA28S. Depicted are results (mean ± SD of triplicate wells; *, p < 0.05, unpaired t-test, CD16^+ ^*versus *CD16^- ^Mo) obtained with matched cells from 2 different healthy donors.

**Figure 3:**
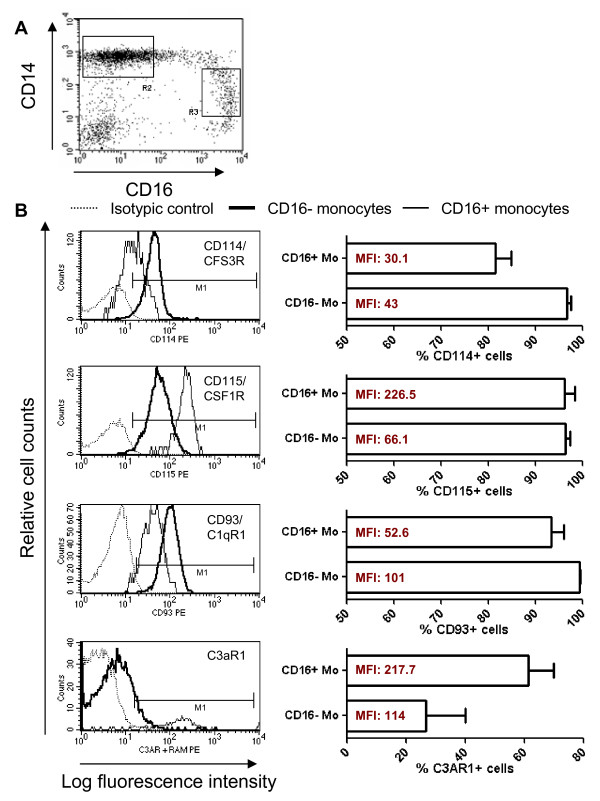
**Differential expression of CD114/CSF3R, CD115/CSF1R, CD93/C1qR1 and C3aR1 on CD16^+ ^and CD16^- ^monocytes**. Freshly isolated PBMC were stained with FITC CD14, PE-Cy5 CD16, and PE CD114, PE CD115, and PE CD93 Abs. The expression of CD3aR1 was detected after staining with unconjugated mouse C3AR1 Ab and PE rat anti-mouse Ab (RAM). CD14^high^CD16^neg ^(R2) and CD14^low^CD16^+ ^(R3) Mo **(A) **were analyzed for expression of CD114, CD115, CD93 and C3aR1 **(B)**. Shown is an overlay histogram from one representative donor of 4 donors examined **(B, left panels) **and graphs showing mean ± SEM for % or MFI of CD114, CD115, CD93 and C3aR1 expression on each Mo subset **(B, right panels)**. (*, Paired t-test p-value < 0.05, CD16^+ ^*versus *CD16^- ^Mo; n = 4).

**Figure 4:**
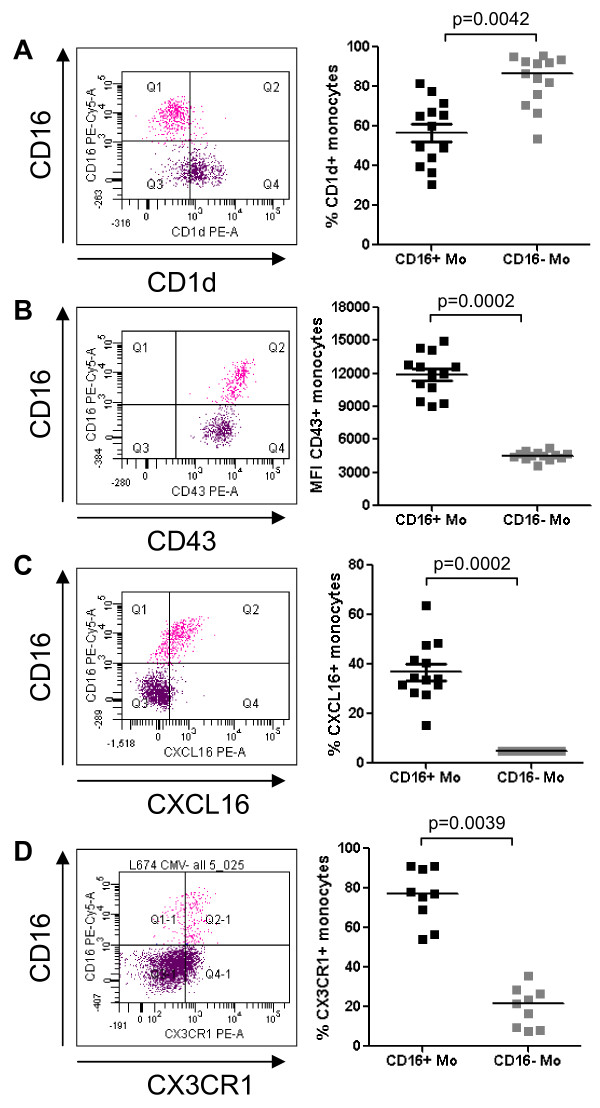
**Differential expression of CD1d, CD43, CXCL16, and CX3CR1 on CD16^+ ^and CD16^- ^monocytes**. Freshly isolated PBMC were stained with Pacific Blue CD3, Alexa700 CD4, FITC CD14, PE-Cy5 CD16, and PE CD1d, PE CD43, PE CXCL16 or PE CX3CR1 Abs. Gated CD3^-^CD4^low^CD14^high^CD16^- ^(CD16^- ^Mo) and CD3^-^CD4^low^CD14^low^CD16^+ ^(CD16^+ ^Mo) cells were analyzed for expression of **(A) **CD1d, **(B) **CD43, **(C) **CXCL16, and **(D) **CX3CR1. Shown are representative dot plots **(left panels) **and results for 9–13 different donors (**right panels**). Paired Wilcoxon signed rank test was used to calculated statistical significance (p < 0.05, CD16^+ ^*versus *CD16^- ^Mo).

Real time RT-PCR was used to quantify expression of nine differentially expressed genes identified by microarray analysis. Results in Figure 2 indicate increased mRNA expression for CD16, C3AR1, ICAM-2, CSF1R, CDKN1C, TNFRSF8, and LTB, and decreased mRNA expression for C1QR1 and CSF3R in CD16^+ ^compared to CD16^- ^Mo (unpaired t-test, p < 0.05, CD16^+ ^*versus *CD16^- ^Mo). Microarray results were also validated at the protein level by flow cytometry analysis. Consistent with the microarray and real time RT-PCR results (Figures 1, 2), FACS analysis demonstrated that CD14^low^CD16^+ ^(gate R3) compared to classical CD14^high^CD16^- ^Mo (gate R2) expressed higher levels of CD115/CSFR1 (M-CSF receptor) and C3AR1, and lower levels of CD114/CSF3R (G-CSF receptor) and CD93/C1qR1 on the cell surface (Figure 3A–B). A third Mo subset with an intermediate phenotype, CD14^high^CD16^+ ^Mo, exhibited intermediate expression levels of CD114 and CD115 and similar levels of CD93 and C3aR1 compared to CD14^low^CD16^+ ^Mo and CD14^high^CD16^- ^Mo, respectively (Additional file 3). In addition, CD16^+ ^compared to CD16^- ^Mo expressed higher levels of CXCL16 and CD43 and lower levels of CD1d (Figure 4A–C). As expected, CX3CR1 was also expressed at higher levels (Figure 4D). Thus, we identified new surface markers that distinguish CD16^- ^Mo (i.e., CD114, CD93, and CD1d) and CD16^+ ^Mo (i.e., CD115, C3AR1, CXCL16, and CD43).

### Biological functions of differentially expressed genes

**Figure 5:**
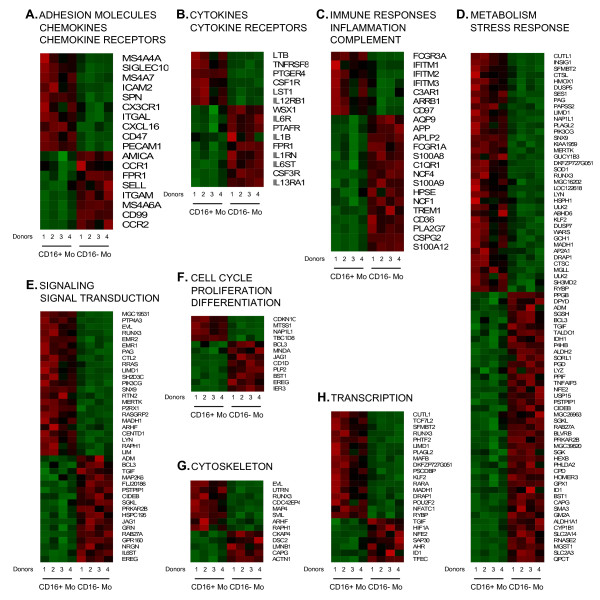
**Biological functions of genes differentially expressed in CD16^+ ^and CD16^- ^monocytes**. Differentially expressed genes were classified based on their biological functions using Gene Ontology as indicated. Heat maps were generated using dChip software and include data from matched CD16^+ ^and CD16^- ^Mo from 4 different individuals. In each heat map, upregulated genes are plotted first followed by downregulated genes. Red and green signify increased and decreased gene expression, respectively.

Differentially expressed genes, corresponding to 250 downregulated and 228 upregulated probe sets in CD16^+ ^compared to CD16^- ^Mo, were classified into eight functional categories using Gene Ontology. Heat maps for biological function categories (Figure 5A–H) showed clear distinctions in patterns of gene expression between the Mo subpopulations for these categories.

#### Adhesion molecules, chemokines, and chemokine receptors

Genes upregulated in CD16^+ ^compared to CD16^- ^Mo included those coding for the tetraspanins MS4A4A and MS4A7, adhesion molecules SIGLEC10, ICAM-2, SPN/CD43, ITGAL/LFA-1/CD11a, CD47, and PCAM1/CD31, chemokine receptor CX3CR1, and chemokine CXCL16 [56]. Genes downregulated in CD16^+ ^compared to CD16^- ^Mo included those coding for the tetraspanin MS4A6A, adhesion molecules ITGAM/CD11b, SELL/L-selectin/CD62L, CD99, and junctional adhesion molecule like (JAML or AMICA) [57], and chemokine receptors CCR1, CCR2, and formyl peptide receptor 1 (FPR1) (Figure 5A). These results identify SIGLEC10, ICAM-2, SPN/CD43, CD47, and CXCL16 as new markers upregulated on CD16^+ ^Mo that are relevant for T cell activation, and FPR1 as a chemokine receptor preferentially expressed on CD16^- ^Mo. In addition, these results indicate the distinct trafficking potential of CD16^+ ^and CD16^- ^Mo (i.e., via CX3CR1 *versus *CCR2, respectively), consistent with previous studies [18-20].

#### Cytokines and cytokine receptors

CD16^+ ^Mo expressed significantly higher levels of mRNA for the cytokines lymphotoxin beta (LTB) and leukocyte specific transcript 1 (LST1) and the cytokine receptors TNF receptor superfamily 8 (TNFRSF8), prostaglandin E receptor 4 (PTGER4), colony stimulating factor 1 receptor (CSF1R; CSF1, controls Mo/MΦ differentiation and function), and IL-12RB1. Genes downregulated in CD16^+ ^Mo included those coding for the cytokine IL-1RA, platelet-activating factor receptor (PTAFR), and IL1B and cytokine receptors IL13RA1, IL27RA (WSX1), colony stimulating factor 3 receptor (CSF3R; CSF3 controls granulocytes differentiation and function), IL6R, and IL6ST/gp130 (a signal transducer shared by many cytokines, including IL-6 and IL-27) (Figure 5B). These results provide further evidence for pro-inflammatory genes upregulated in CD16^+ ^Mo (e.g., HMOX1 and SOD1) [12] and suggest the ability of CD16^+ ^and CD16^- ^Mo to respond to distinct cytokines including IL-12 [58] and IL-13 [59] and IL-6 and IL-27 [60,61], respectively, which has potential implications for Th1 and Th2 polarization of immune responses. In addition, these results identify CSF1R and CSF3R as new markers for CD16^+ ^and CD16^- ^Mo, respectively, with potential implications for their differentiation fate *in vivo*.

#### Immune responses, inflammation, and complement

CD16^+ ^Mo expressed significantly higher levels of mRNA for the low affinity Fcγ receptor FCGR3A/CD16, IFN-γ-induced surface molecules IFITM1, IFITM2 and IFITM3, complement receptor C3AR1, arrestin beta 1 (ARRB1, which contributes to desensitization of G-protein-coupled receptors), and CD97 (receptor for complement decay accelerating factor, DAF/CD55). Genes downregulated in CD16^+ ^Mo included those coding for the high affinity Fcγ receptor FCGR1A/CD64, complement receptor C1QR1, Ca binding proteins S100A12, S100A9, and S100A8, phospholipase A2, group VII (PLA2G7), Ig superfamily receptor TREM1, neutrophil cytosolic factors NCF1 and NCF4, heparanase (HPSE), chondroitin sulfate proteoglycan 2 (versican, CSPG2), amyloid beta (A4) precursor-like protein 2 (APLP2) involved in turnover of MHC Class I molecules [62], amyloid beta (A4) precursor protein (APP), and aquaporin AQP9, which plays a role in immunological response and bactericidal activity (Figure 5C). These results identify complement-related molecules C3AR1 and CD97 as new markers for CD16^+ ^Mo, and C1QR1 as a new marker for CD16^- ^Mo.

#### Metabolism and stress response

Transcripts upregulated in CD16^+ ^Mo included genes related to protein synthesis (i.e., tryptophanyl-tRNA synthetase (WARS)), protein catabolism (*i.e.*, cathepsin L (CTSL), cathepsin C (CTSC)), stress responses (*i.e.*, heme oxygenase (decycling) 1 (HMOX1), superoxide dismutase 1 (SOD1), heat shock 105 kDa/110 kDa protein 1 (HSPH1), and monoglyceride lipase (MGLL)), and insulin induced gene 1 (INSIG1). Genes downregulated in CD16^+ ^Mo included those coding for enzymes related to protein metabolism (i.e., glutaminyl-peptide cyclotransferase (QPCT), microsomal glutathione S-transferase 1 (MGST1), carboxypeptidase D (CPD), ubiquitin specific protease 15 (USP15), peptidylprolyl isomerase F (cyclophilin F; PPIF), and N-sulfoglucosamine sulfohydrolase (sulfamidase)/SGSH), stress responses (i.e., aldehyde dehydrogenase 1 family, member A1 (ALDH1A1), cytochrome P450, family 1, subfamily B, polypeptide 1 (CYP1B1), glutathione peroxidase 1 (GPX1), tumor necrosis factor, alpha-induced protein 3 (TNFAIP3), and aldehyde dehydrogenase 2 family (ALDH2)) and other enzymatic processes (i.e., ribonuclease, RNase A family, 2 (RNASE2) and lysozyme (LYZ)) (Figure 5D). Differential expression of SOD1 and GPX1 in CD16^+ ^and CD16^- ^Mo, respectively, indicates a distinct antioxidant enzymatic defense system in these Mo subsets. The upregulation of WARS expression in CD16^+ ^Mo suggests increased potential protein synthesis [63], while CTSL and CTSC upregulation may indicate increased potential antigenic processing and antigen presentation capacity [64].

#### Signaling and signal transduction

CD16^+ ^Mo expressed significantly higher levels of transcripts for a large number of genes involved in signal transduction including the protein tyrosine phosphatase type IVA, member 3 (PTP4A3) and phosphoinositide-3-kinase, catalytic, gamma polypeptide (PIK3CG, a crucial signaling molecule required for macrophage accumulation in inflammation [65]). Genes downregulated in CD16^+ ^Mo included those coding for the interleukin 6 signal transducer (IL6ST), G protein-coupled receptor 160 (GPR160), jagged 1 (JAG1, the ligand for the receptor notch 1), protein kinase, cAMP-dependent, regulatory, type II, beta (PRKAR2B), the CD2 binding protein proline-serine-threonine phosphatase interacting protein 1 (PSTPIP1), and mitogen-activated protein kinase kinase 6 (MAP2K6) (Figure 5E). Thus, CD16^+ ^and CD16^- ^Mo exhibit distinct signaling pathway activation, indicating a distinct activation/differentiation history *in vivo*.

#### Cell cycle, proliferation and differentiation

CD16^+ ^Mo were distinguished from CD16^- ^Mo by upregulation of the cell cycle related genes cyclin-dependent kinase inhibitor 1C (CDKN1C, p27, or KIP2, which is a negative regulator of cell proliferation [66] induced by TGF-β [67]), and metastasis suppressor 1 (MTSS1, a transcript involved in cytoskeleton organization missing in metastasis [68]). CD16^- ^Mo preferentially expressed mRNA for genes encoding the CD1d antigen (member of the MHC family that mediates presentation of primarily lipid/glycolipid antigens to T cells), and myeloid cell nuclear differentiation antigen (MNDA, which is expressed in human monocytes and granulocytes and earlier stage cells in the myeloid lineage [69]) (Figure 5F). These results provide evidence that CD16^+ ^Mo represent a more advanced stage of differentiation compared to CD16^- ^Mo.

#### Cytoskeleton

CD16^+ ^were distinguished from CD16^- ^Mo by expression of a series of genes related to the cytoskeleton showing higher expression in CD16^+ ^Mo including CDC42 effector protein (Rho GTPase binding) 4 (CDC42EP4), microtubule-associated protein 4 (MAP4), and supervillin (SVIL) and in CD16^- ^Mo including actinin, alpha 1 (ACTN1). (Figure 5G).

#### Transcription factors

CD16^+ ^Mo expressed significantly higher levels of mRNA for several transcriptional factor genes including the macrophage transcription factor v-maf musculoaponeurotic fibrosarcoma oncogene homolog B (MafB, an essential determinant of the monocytic program in hematopoietic cells [7,70,71]), the pleckstrin homology, Sec7, and coiled-coil protein-binding protein (PSCDBP or CYBR, a cytohesin-1-binding protein expressed in NK cells stimulated with IL-2 and IL-12 that plays a role in integrin-mediated cell adhesion [72]), the Kruppel-like factor 2 (KLF2, reported to license mature T-cells for trafficking from the thymus and recirculation through secondary lymphoid tissues [73]), and retinoic acid receptor, alpha (RARA, expressed in dendritic cells [74] and involved in myeloid differentiation [75] and imprinting for gut homing [76,77]). In contrast, CD16^- ^Mo preferentially expressed the aryl hydrocarbon receptor (AHR, a ligand dependent E3 ubiquitin ligase [78] and modulator of anti-viral immunity [79]) transcript (Figure 5H). Both CD16^+ ^and CD16^- ^Mo lacked PU.1 expression, a transcription factor upregulated in DC [71], as demonstrated by microarray analysis and RT-PCR (data not shown). Thus, CD16^+ ^and CD16^- ^Mo express distinct transcription factors that may differentially regulate biological functions *in vivo*.

### Gene set enrichment analysis (GSEA)

To extract further meaning from differentially expressed genes in CD16^+ ^and CD16^- ^Mo, GSEA, a knowledge based approach for interpreting genome-wide expression profiles [54], was applied to test for sets of genes that share common biological functions. Enrichment scores (ES), nominal p-values, false discovery rate (FDR), and family wise-error rate (FWER) values were generated for a large number of gene sets for GSEA available on the Molecular Signatures Database (MSigDB) of the Broad Institute. One gene set was significantly enriched in CD16^- ^compared to CD16^+ ^Mo: HADDAD_HPCLYMPHO_ENRICHED (p < 0.001; both FDR q-value and FWER p-value- < 0.2). According to MSigDB, this set includes genes enriched in CD45RA^hi^Lin^-^CD10^+ ^versus CD45RA^int^CD7^- ^and CD45RA^hi^CD7^hi ^hematopoietic progenitor cells [80]. Four uncharacterized open reading frames upregulated in hematopoietic progenitor cells (i.e., C18ORF1, CYORF15B, C6ORF62, and C6ORF111) [80] were significantly enriched in CD16^- ^Mo.

GSEA also identified several gene sets relatively enriched in CD16^+ ^or CD16^- ^Mo, but with a lower statistical significance likely related to the limited number of samples (p < 0.001 and FDR = 1). These analyses showed that CD16^+ ^Mo were enriched in genes related to NK cell mediated toxicity (i.e., FcγRIIIA, PIK3CG, NFATC1, ITGAL, and ICAM-2), inositol phosphate metabolism (i.e., PIK3CG), actin binding (i.e., MTSS1, COTL1, and SVIL), and oxidative stress (i.e., CDKN1C, ETS1, CD47, LYN, and VIL2). In contrast, CD16^- ^Mo were enriched in genes related to hematopoietic cell lineage (i.e., CD1d, IL1β, FcγRIA, ITGAM, CSF3R, CD36, and CD14), receptor mediated endocytosis (i.e., SORL1, STAB1, FCGR1A, and CD14), arginine and proline metabolism (i.e., ALDH2, P4HB, and ALDH1A1), nontypable *Haemophilus influenzae *(NTHi) pathway (i.e., IL1B and MAP2K6), and lipid binding molecules (i.e., CD1D, PTAFR, ALDH1A1, and PLA2G7). Overall, these results provide new insights into the developmental relationship between CD16^+ ^and CD16^- ^Mo, with CD16^- ^Mo being more closely related to hematopoietic progenitor cells and having higher endocytosis activity, while CD16^+ ^Mo being at a more advanced stage of Mo differentiation with more effector functions related to antigen presentation, migration, and cytotoxicity.

### Pattern recognition receptor expression in CD16^+ ^and CD16^- ^monocytes

Both Mo subsets expressed TLR1, TLR2, TLR4, TLR5, and TLR8 but not TLR3, TLR6, TLR7, TLR9, and TLR10 mRNA (data not shown). Considering that CD16^+ ^Mo express low levels of the LPS co-receptor, CD14 [11], and that TLR stimulation was previously implicated in myeloid differentiation [26,81], we tested whether genes associated with TLR pathway were upregulated in these cells. However, GSEA rejected this hypothesis. Nonetheless, microarray results demonstrated slight downregulation of TLR2, TLR4, TLR5, and TLR8, together with slight upregulation of MyD88, a key adaptor for these TLRs [82], in CD16^+ ^compared to CD16^- ^Mo

### Potential imprinting for non-skin homing in CD16^+ ^monocytes

**Figure 6:**
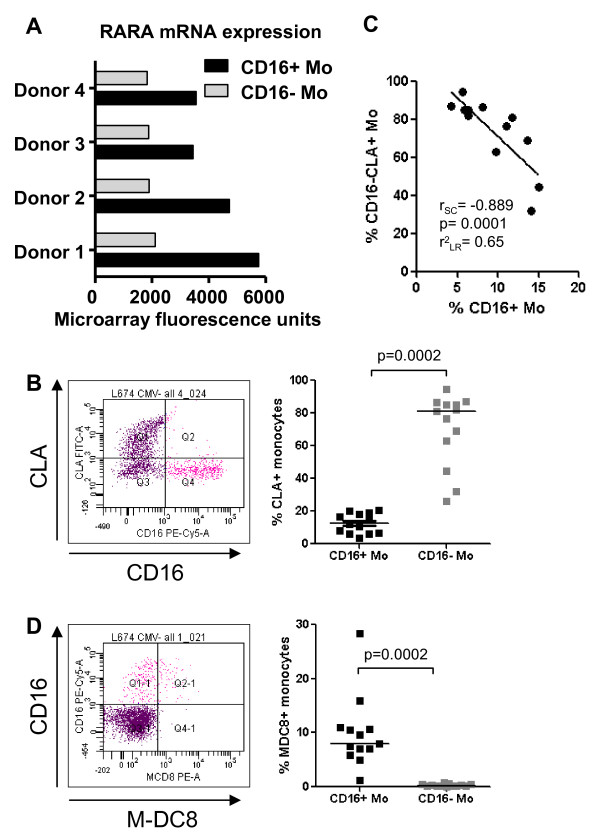
**Differential expression of RARA mRNA and PSGL-1 epitopes CLA and M-DC8 on CD16^+ ^and CD16^- ^monocytes**. **(A) **Differential expression of RARA mRNA in CD16^+ ^and CD16^- ^Mo was extracted from microarray data set results and expressed as relative fluorescence units. **(B-D) **Freshly isolated PBMC were stained with Pacific Blue CD3, Alexa700 CD4, PE-Cy5 CD16, FITC CLA and PE M-DC8 Abs. Gated CD3^-^CD4^low^CD14^high^CD16^- ^(CD16^- ^Mo) and CD3^-^CD4^low^CD14^low^CD16^+ ^(CD16^+ ^Mo) cells were analyzed for expression of CLA **(C) **and M-DC8 **(D)**. **(B and D) **Shown are representative dot plots **(left panels) **and results for 13 different donors **(right panels)**. Paired Wilcoxon signed rank test was used to calculated statistical significance (p < 0.05). **(B) **Spearman correlation (r and p values) and linear regression (r^2 ^value) were calculated to examine the relationship between the frequency of CD16^-^CLA^+ ^Mo and CD16^+ ^Mo.

We demonstrated increased expression of RARA mRNA in CD16^+ ^compared to CD16^- ^Mo (Figure 5H and 6A). SLP-76 (Src-homology 2 domain-containing leukocyte specific phosphoprotein of 76 kDa), a RA-induced target [75], was significantly upregulated in CD16^+ ^compared to CD16^- ^Mo (6363 ± 611 versus 3882 ± 565; CD16^+^/CD16^- ^ratio 1.64; p = 0.005), indicative of RARA pathway activation in CD16^+ ^Mo. Activation of the RARA transcription factor pathway leads to loss of skin homing potential in lymphocytes *via *downregulation of the cutaneous lymphocyte-associated antigen (CLA, an epitope on PSGL-1) [83] and imprinting for mucosal homing [77]. CLA expression was quantified on CD16^+ ^and CD16^- ^Mo by FACS on PBMC from healthy individuals. CLA expression was undetectable on all CD16^+ ^Mo and a fraction of CD16^- ^Mo (Figure 6B). The frequency of CLA^+^CD16^- ^Mo was negatively correlated with the frequency of CD16^+ ^Mo (Figure 6C). CD16^+ ^Mo express M-DC8 (an epitope on PSGL-1) [13], which was previously reported to be expressed by a subset of mucosal DC [84] (Figure 6D). CX3CR1 expression on CD16^+ ^Mo (Figure 4D) [18] may contribute to recruitment of these cells into CX3CL1 expressing tissues including the gut. These results indicate that CD16^+ ^Mo, similar to RA-stimulated T-cells [77], lack expression of the skin-homing addressin CLA and therefore are potentially imprinted for non-skin homing. Because retinoic acid (RA) is an important factor driving myeloid differentiation [75], this reprogramming of CD16^+ ^Mo homing potential may be in part a consequence of RARA pathway activation.

## Discussion

In this study, we define transcriptional profiles of human CD16^+ ^and CD16^- ^monocytes (Mo) and provide new insights into their developmental relationship and biological functions. Despite remarkable transcriptional similarity (approximately 83%), a significant number of transcripts were differentially expressed (n = 2,759), with 228 and 250 >2-fold upregulated and downregulated, respectively, in CD16^+ ^compared to CD16^- ^Mo. Differentially expressed genes related to cell-to-cell adhesion and trafficking, immune responses and inflammation, metabolism and stress response, signaling and signal transduction, cell cycle, proliferation, and differentiation, cytoskeleton, and regulation of transcription. Gene set enrichment analysis (GSEA) demonstrated that CD16^+ ^Mo are enriched in genes related to NK-mediated cytotoxicity, inositol phosphate metabolism, actin binding, and oxidative stress, while CD16^- ^Mo are enriched in genes related to hematopoietic cell lineage, receptor-mediated endocytosis, arginine and proline metabolism, NTHi pathway, and lipid binding. The transcriptional profiles suggest that CD16^+ ^and CD16^- ^Mo subsets originate from a common myeloid precursor, with CD16^+ ^Mo being at a more advanced stage of myeloid differentiation and having distinct biological functions *in vivo*.

Previous studies in mice provide evidence for a developmental relationship between Ly6C^high^CCR2^high^Gr1^+^CX3CR1^low ^and Ly6C^low^CCR2^low^Gr1^-^CX3CR1^high ^Mo (homologs of human CD16^- ^and CD16^+ ^Mo, respectively), with Ly6C^low^CCR2^low^Gr1^-^CX3CR1^high ^Mo being more mature and derived from Ly6C^high^CCR2^high^Gr1^+^CX3CR1^low ^Mo [5,41,85]. Likewise, studies on human Mo demonstrated the ability of CD16^-^CX3CR1^low ^Mo to differentiate into CD16^+^CX3CR1^high ^Mo upon stimulation with TGF-β, IL-10, M-CSF, or CCL2 [17,43-45]. Our comparative transcriptome analysis provides further evidence for the idea that CD16^- ^Mo originate from a common granulocyte-macrophage (GM) precursor and give rise to CD16^+ ^Mo, which are more closely related to macrophages (MΦ) and dendritic cells (DC). CD16^- ^Mo preferentially expressed granulocyte-associated transcripts (*i.e.*, CSF3R, formyl peptide receptor 1 (FPR1), the calgranulins S100A8, S100A9, and S100A12), and myeloid markers (*i.e.*, CD14, MNDA, TREM-1, CD1d, and C1qR1/CD93), together with transcripts suggesting an increased potential for receptor-mediated endocytosis via molecules such as CD14 and FCGR1A/CD64 [85,86]. In contrast, CD16^+ ^Mo preferentially expressed MΦ (i.e., CSF1R/CD115, MafB, EGF module-containing mucin-like hormone receptor (EMR)1-3, CD97, and C3aR) [86] and DC markers (*i.e.*, SIGLEC10, CD43, CXCL16, and RARA) [56,74,87,88]. CD16^+ ^Mo expressed higher levels of transcripts encoding the cysteine protease cathepsin L (CTSL), which contributes to phagocytic-endocytic proteolysis in DC for subsequent antigen presentation [64]. Upregulation of transcripts encoding dipeptidyl-peptidase I, CTSC [89] may further enhance antigen processing by CD16^+ ^Mo or DC derived from these cells. Although some studies classified CD16^+ ^Mo as DC based on their increased antigen presenting ability [14-16] and transcriptional profile similarities [16], a recent compendium analysis of transcriptional profiles demonstrated that CD16^+^HLA-DR^+ ^cells are more closely linked to myeloid CD14^+ ^cells than to DC subsets in peripheral blood [90]. Our results demonstrate that CD16^+ ^Mo share approximately 83% of their transcripts with CD16^- ^Mo, supporting the idea that these two Mo subsets are developmentally related.

Recruitment of CD16^+ ^and CD16^- ^Mo into tissues is mediated *via *distinct molecular mechanisms [7,8]. Our gene expression analysis confirms differential expression of adhesion molecules and chemokine receptors previously reported to be preferentially expressed on CD16^+ ^Mo (i.e., LFA-1, PECAM/CD31, CX3CR1) and CD16^- ^Mo (i.e., CCR1, CCR2, and L-selectin/CD62L) [18-20]. We also identified new cell surface markers and other molecules that are differentially expressed in these Mo subsets and may influence their trafficking and migration into tissues. The tetraspanins MS4A4A and MS4A7, adhesion molecules SIGLEC10 and ICAM-2, and membrane-bound chemokine CXCL16 [56] were preferentially expressed by CD16^+ ^Mo, whereas the tetraspanin MS4A6A, adhesion molecules CD99 and junctional adhesion molecule like (JAML or AMICA) [57], and chemokine receptor FPR1 were preferentially expressed by CD16^- ^Mo. CD31 and CD99 are involved in distinct steps of Mo transendothelial migration [91,92]. Expression of CXCL16, a chemokine expressed by DC [56], on the surface of CD16^+ ^Mo may facilitate interaction with CXCR6^+ ^cells (i.e., NKT and activated CD4^+ ^and CD8^+ ^T-cells [56]) and retention of CXCR6^+ ^cells in tissues. Similar to mouse and rat CCR2^low^CX3CR1^high ^Mo [37,41], CD16^+ ^Mo expressed higher levels of SPN/CD43 (sialophorin, leukosialin, large sialoglycoprotein or gp115), a ligand for ICAM-1 [93,94], and the macrophage adhesion receptor sialoadhesin (Siglec-1) [95]. CD43 has both adhesive and anti-adhesive properties [96], mediates DC maturation [88], and contributes to regulation of immunological synapse formation [97]. CD47, a receptor for thrombospondin-1 (TSP-1), is preferentially expressed by CD16^+ ^Mo. CD47 ligation selectively inhibits the development of human naive T cells into Th1 effectors by decreasing IL-12 and TNF-α production by Mo-derived DC [98,99]. Consistent with these findings, CD16^- ^and CD16^+ ^Mo may induce Th1 and Th2-like differentiation, respectively [100]. However, CD16^+ ^Mo express IL-12RB1, which favors Th1 polarization [58], whereas CD16^- ^Mo express receptors for the Th2 cytokines IL-6 and IL-13 [59] and the anti-Th1 cytokine, IL-27 [60,61]. Accordingly, the influence of CD16^+ ^and CD16^- ^Mo on Th1 versus Th2 polarization of immune responses is likely to be highly dependent on the local microenvironment within tissues.

CD16^+ ^Mo expressed high levels of transcripts for RARA, which controls transcription of genes involved in cell trafficking and mucosal homing. RA imprints lymphocytes with non-skin mucosal homing properties by decreasing cutaneous lymphocyte-associated antigen (CLA, an epitope on PSGL-1) expression [83] and increasing expression of CCR9 and integrin beta 7, two mucosal addressins [77]. RA also controls reciprocal differentiation of Th17 and regulatory T cells [101], modulates myeloid gene expression and differentiation [75], and regulates survival and antigen presentation by DC [74]. Consistent with our hypothesis that the RARA pathway is activated in CD16^+ ^Mo, we demonstrated CLA downregulation on these cells, together with upregulation of two RA-induced targets: SLP-76 [75] and CXCL16 [102]. RA induces mucosal-type DC, which produce TGF-β and thereby imprints T-cells for gut homing by inducing CCR9 and integrin beta 7 [76]. CD16^+ ^Mo-derived MΦ and DC constitutively produce TGF-β [23,100], but whether they also instruct T-cells for gut homing remains to be determined.

KLF2 mRNA is expressed at very high levels and significantly upregulated in CD16^+ ^compared to CD16^- ^Mo. KLF2 belongs to a family of zinc-finger transcription factors that is induced by PI3K signaling [103] and controls expression of several genes including those coding for CD62L, CCR7, integrin beta7, sphingosine-1-phosphate receptor (S1PR1) [73], and lymphotoxin beta [104]. CCR7 and CD62L are essential for migration into lymph nodes, S1P1 regulates T-cell thymic egress and recirculation [105], and integrin beta 7 mediates cell recruitment into Peyer's patches and mesenteric lymph nodes [106]. Together, these findings raise the possibility that preferential expression of KLF2 in CD16^+ ^Mo may confer an increased potential for trafficking.

CD16^+ ^compared to CD16^- ^Mo express very high levels of transcripts for cyclin-dependent kinase inhibitor 1C (CDKN1C or p57/KIP2) (18.4-fold increase) and metastasis suppressor 1 MTSS1 (5.7-fold increase). CDKN1C is a potent inhibitor of several G1 cyclin-dependent kinase (cdk) complexes, and negative regulator of G1/S cell cycle transition and cell proliferation [66]. CDKN1C [66] and MTSS1 [68] are candidate tumor suppressor genes, and their high expression is consistent with the inability of Mo to proliferate [7]. CDKN1C is induced by TGF-β [67], a cytokine known to induce CD16^+ ^Mo differentiation [17,43]. Thus, our results are consistent with a potential link between TGF-β pathway activation and CD16^+ ^Mo differentiation *in vivo*.

Several transcripts related to cell activation were upregulated in CD16^+ ^Mo including LTB, TNFRSF8, leukocyte specific transcript 1 (LST1), IFITM1-3, HMOX1, superoxide dismutase-1 (SOD-1), tryptophanyl tRNA synthetase (WARS), and monoglyceride lipase (MGLL), indicating increased activation of CD16^+ ^compared with CD16^- ^Mo. LST1 [107], HMOX1 [108], SOD-1, and WARS are induced by stimulation with lipopolysaccharide [109]. The role of LST1 in immune regulation remains elusive. HMOX1 modulates Mo inflammatory responsiveness by decreasing LPS-induced TNF and IL-1β expression [108]. WARS and indoleamine 2,3-dioxygenase (IDO) are responsible for tryptophan use in protein synthesis and degradation, respectively [63]. WARS was identified as a molecular marker for Mo differentiation into MΦ [110] and DC [111]. These results suggest increased activation of CD16^+ ^compared to CD16^- ^Mo *in vivo*.

The CD16^+ ^Mo subset includes two subsets with distinct levels of CD14 expression: CD14^high^CD16^+ ^and CD14^low^CD16^+ ^[11,112]. CD14^high^CD16^+ ^Mo exhibit a phenotype intermediate between that of CD14^high^CD16^neg ^and CD14^low^CD16^+ ^Mo in terms of adhesion molecule (*e.g.*, CL62L) and chemokine receptor expression (*e.g.*, CCR2, CXCR2, and CX3CR1) [18]. Both CD14^high^CD16^+ ^and CD14^low^CD16^+ ^Mo contributed to the transcriptional profile of CD16^+ ^Mo in this study. The expression of some genes we identified as markers for CD16^+ ^Mo may be distinct on CD14^high^CD16^+ ^and CD14^low^CD16^+ ^Mo. Consistent with this prediction, we demonstrated intermediate expression of CD115 and CD114 on CD14^high^CD16^+ ^Mo compared to CD14^high^CD16^neg ^and CD14^low^CD16^+ ^Mo, and high expression of CD93 and C3aR1, similar to that on CD14^high^CD16^- ^and CD14^low^CD16^+ ^Mo, respectively (Additional file 3). These findings suggest a developmental relationship between these Mo subsets in which CD14^high^CD16^neg ^Mo, CD14^high^CD16^+ ^Mo, and CD14^low^CD16^+ ^Mo represent sequential stages of monocyte differentiation [41].

## Conclusion

Comparative transcriptome analysis of CD16^+ ^and CD16^- ^Mo indicates that CD16^+ ^Mo represent a more advanced stage of myeloid differentiation with a more MΦ – and DC-like transcription program, whereas CD16^- ^Mo are more closely related to a common myeloid precursor. Given the ability of CD16^+ ^and CD16^- ^Mo to be recruited into specific tissues *via *distinct mechanisms, these Mo subsets are likely to give rise to DC and MΦ subpopulations with distinct phenotypes and roles in immunity and disease pathogenesis. Further studies to characterize phenotypic differences between CD16^+ ^and CD16^- ^Mo-derived DC and MΦ are relevant for development of DC-based vaccines, and will also provide a better understanding of their functional roles in immune responses, inflammation, and disease pathogenesis.

## Competing interests

The authors declare that they have no competing interests.

## Authors' contributions

PA designed and performed experiments, analyzed and interpreted data, prepared graphics, and wrote the manuscript. VM generated heat maps, performed statistical analysis for differentially expressed genes, and drafted the Methods for Figure 5. KYL and XZ performed statistical analysis of microarray data and drafted the Methods and Results. KYL classified genes based on biological functions and performed GSEA. VSW and AG carried out experiments in Figures 4 and 6 and drafted the Methods and Results. DG conceived the study, designed experiments, analyzed and interpreted data, and wrote the manuscript. All authors revised and gave final approval for publication of the manuscript.

## Supplementary Material

Additional file 1 **Table S1. Genes upregulated in CD16^+ ^compared to CD16^- ^monocytes**. Calculation of expression ratios for the 2,759 differentially expressed probe sets showed upregulation of 228 probe sets (corresponding to 153 genes and 19 unknown transcribed sequences) in CD16^+ ^compared to CD16^- ^Mo (cut-off 2-fold; p < 0.05). Click here for file

Additional file 2 **Table S2. Genes downregulated in CD16^+ ^compared to CD16^- ^monocytes**. Calculation of expression ratios for the 2,759 differentially expressed probe sets showed downregulation of 250 probe sets (corresponding to 166 genes and 23 unknown transcribed sequences) in CD16^+ ^compared to CD16^- ^Mo (cut-off 2-fold; p < 0.05). Click here for file

Additional file 3 **Figure S1. Differential expression of CD114/CSF3R, CD115/CSF1R, CD93/C1qR1 and C3aR1 on CD14^high^CD16^-^, CD14^high^CD16^+^, and CD14^low^CD16^+ ^monocytes**. Freshly isolated PBMC were stained with FITC CD14, PE-Cy5 CD16, and PE CD114, PE CD115, and PE CD93 Abs. The expression of CD3aR1 was detected after staining with unconjugated mouse C3AR1 Ab and PE rat anti-mouse Ab (RAM). CD14^high^CD16^neg ^(R2), CD14^high^CD16^+ ^(R3) and CD14^low^CD16^+ ^(R4) Mo **(A) **were analyzed for expression of CD114, CD115, CD93 and C3aR1 **(B)**. Shown is an overlay histogram from one representative donor of 4 donors examined **(B, left panels) **and graphs showing mean ± SEM for % or MFI of CD114, CD115, CD93, and C3aR1 expression on each Mo subset **(B, right panels)**. (*, Paired t-test p-values < 0.05, CD16^+ ^*versus *CD16^- ^Mo; n = 4). Click here for file
