# Supplementary material for: Transcriptional profiling reveals developmental relationship and distinct biological functions of CD16+ and CD16- monocyte subsets
Source: BMC Genomics. 2009 Aug 27;10:403. doi: 10.1186/1471-2164-10-403 (PMC2741492; doi:10.1186/1471-2164-10-403)
Supplement: Additional file 1 — Table S1. Genes upregulated in CD16+ compared to CD16- monocytes. Calculation of expression ratios for the 2,759 differentially expressed probe sets showed upregulation of 228 probe sets (corresponding to 153 genes and 19 unknown transcribed sequences) in CD16+ compared to CD16- Mo (cut-off 2-fold; p < 0.05). [file 1471-2164-10-403-S1.pdf]

Supplemental Table 1 - Genes upregulated in CD16+ monocytes

Known genes (n=153)

GeneSymbol	ratio	p-value	ProbeSetN	ProbeSetID	GeneTitle
FCGR3A	20,1	0,000	3595	204006_s_at	Fc fragment of IgG, low affinity IIIa, receptor for (CD16)
CDKN1C	18,4	0,000	12791	213348_at	cyclin-dependent kinase inhibitor 1C (p57, Kip2)
MGC19531	6,7	0,000	475	200885_at	hypothetical protein MGC19531
CUTL1	5,8	0,000	1957	202367_at	cut-like 1, CCAAT displacement protein (Drosophila)
MTSS1	5,7	0,000	2628	203037_s_at	metastasis suppressor 1
EAT2	5,7	0,000	45441	1553177_at	SH2 domain-containing molecule EAT2
MS4A4A	5,5	0,000	19033	219607_s_at	membrane-spanning 4-domains, subfamily A, member 4
CHST2	5,4	0,000	3510	203921_at	carbohydrate (N-acetylglucosamine-6-O) sulfotransferase 2
CYFIP2	5,3	0,000	15220	215785_s_at	cytoplasmic FMR1 interacting protein 2
TCF7L2	5,1	0,000	12209	212762_s_at	transcription factor 7-like 2 (T-cell specific, HMG-box)
PTP4A3	5,0	0,000	9245	209695_at	protein tyrosine phosphatase type IVA, member 3
INSIG1	5,0	0,000	1215	201625_s_at	insulin induced gene 1
SIGLEC10	4,8	0,000	45167	1552807_a_at	sialic acid binding Ig-like lectin 10
IFITM1	4,5	0,026	13463	214022_s_at	interferon induced transmembrane protein 1 (9-27)
SFMBT2	4,4	0,000	27079	227210_at	Scm-like with four mbt domains 2
SGPP1	4,2	0,000	20693	221268_s_at	sphingosine-1-phosphate phosphatase 1
MS4A7	4,1	0,000	23235	223344_s_at	membrane-spanning 4-domains, subfamily A, member 7
CTSL	4,0	0,000	1677	202087_s_at	cathepsin L
EVL	3,7	0,000	17265	217838_s_at	Enah/Vasp-like
TAGLN	3,7	0,000	5136	205547_s_at	transgelin
RUNX3	3,7	0,000	3787	204198_s_at	runt-related transcription factor 3
SAMSN1	3,6	0,000	54073	1569599_at	SAM domain, SH3 domain and nuclear localisation signals, 1
TM7SF1	3,6	0,000	3726	204137_at	transmembrane 7 superfamily member 1 (upregulated in kidney)
HMOX1	3,5	0,000	3254	203665_at	heme oxygenase (decycling) 1
DUSP5	3,5	0,000	9012	209457_at	dual specificity phosphatase 5
ADA	3,5	0,000	4228	204639_at	adenosine deaminase
EMR2	3,5	0,000	7193	207610_s_at	egf-like module containing, mucin-like, hormone receptor-like 2
SES1	3,4	0,000	1052	201462_at	secernin 1
EMR1	3,4	0,000	6699	207111_at	egf-like module containing, mucin-like, hormone receptor-like 1
PAG	3,3	0,000	25497	225626_at	phosphoprotein associated with glycosphingolipid-enriched microdomains
CTL2	3,3	0,000	24482	224609_at	CTL2 gene
RRAS	3,3	0,000	12094	212647_at	related RAS viral (r-ras) oncogene homolog
PHTF2	3,2	0,000	9330	209780_at	putative homeodomain transcription factor 2
LTB	3,2	0,000	6925	207339_s_at	lymphotoxin beta (TNF superfamily, member 3)
FLJ14299	3,2	0,025	22653	222760_at	hypothetical protein FLJ14299
C20orf55	3,2	0,000	26454	226584_s_at	chromosome 20 open reading frame 55
MLP	3,2	0,000	234	200644_at	MARCKS-like protein
PAPSS2	3,2	0,000	2651	203060_s_at	3'-phosphoadenosine 5'-phosphosulfate synthase 2

LIMD1	3,2	0,000	22655	222762_x_at	LIM domains containing 1
NAP1L1	3,1	0,000	13306	213864_s_at	nucleosome assembly protein 1-like 1
PLAGL2	3,0	0,000	2516	202925_s_at	pleiomorphic adenoma gene-like 2
SH2D3C	2,9	0,000	26542	226673_at	SH2 domain containing 3C
TNFRSF8	2,9	0,000	6317	206729_at	tumor necrosis factor receptor superfamily, member 8
PTGER4	2,9	0,000	4486	204897_at	prostaglandin E receptor 4 (subtype EP4)
MDM1	2,9	0,000	13203	213761_at	nuclear protein double minute 1
PIK3CG	2,8	0,000	5958	206370_at	phosphoinositide-3-kinase, catalytic, gamma polypeptide
SNX9	2,8	0,000	22920	223027_at	sorting nexin 9
SPRED1	2,8	0,019	26706	226837_at	sprouty-related, EVH1 domain containing 1
CSF1R	2,8	0,000	2695	203104_at	colony stimulating factor 1 receptor
SNFT	2,8	0,000	19784	220358_at	Jun dimerization protein p21SNFT
COTL1	2,8	0,000	47743	1556346_at	coactosin-like 1 (Dictyostelium)
CHST7	2,8	0,000	6344	206756_at	carbohydrate (N-acetylglucosamine 6-O) sulfotransferase 7
CD79B	2,8	0,000	4886	205297_s_at	CD79B antigen (immunoglobulin-associated beta)
FER1L3	2,8	0,022	11315	211864_s_at	fer-1-like 3, myoferlin (C. elegans)
DKFZP727G051	2,8	0,000	27080	227211_at	DKFZP727G051 protein
JFC1	2,7	0,000	27003	227134_at	NADPH oxidase-related, C2 domain-containing protein
CLN6	2,7	0,022	22223	64408_s_at	ceroid-lipofuscinosis, neuronal 6, late infantile, variant
SLC2A6	2,7	0,000	19517	220091_at	solute carrier family 2 (facilitated glucose transporter), member 6
UTRN	2,7	0,000	24964	225093_at	utrophin (homologous to dystrophin)
FMNL2	2,6	0,000	26054	226184_at	formin-like 2
KIAA1959	2,6	0,000	38325	238462_at	nm23-phosphorylated unknown substrate
ICAM2	2,6	0,000	13062	213620_s_at	intercellular adhesion molecule 2
TBC1D8	2,6	0,000	4115	204526_s_at	TBC1 domain family, member 8 (with GRAM domain)
RTN2	2,6	0,000	21869	34408_at	reticulon 2
MERTK	2,6	0,000	5617	206028_s_at	c-mer proto-oncogene tyrosine kinase
SPN	2,5	0,000	5645	206057_x_at	sialophorin (gpL115, leukosialin, CD43)
MAFB	2,5	0,000	22563	222670_s_at	v-maf musculoaponeurotic fibrosarcoma oncogene homolog B (avian)
GUCY1B3	2,5	0,000	3406	203817_at	guanylate cyclase 1, soluble, beta 3
DRCTNNB1A	2,5	0,000	27108	227239_at	down-regulated by Ctnnb1, a
IFITM2	2,5	0,000	905	201315_x_at	interferon induced transmembrane protein 2 (1-8D)
E2IG5	2,5	0,000	24226	224345_x_at	growth and transformation-dependent protein
P2RX1	2,5	0,000	9941	210401_at	purinergic receptor P2X, ligand-gated ion channel, 1
SOD1	2,5	0,000	232	200642_at	superoxide dismutase 1, soluble (amyotrophic lateral sclerosis 1 (adult))
IFITM3	2,5	0,022	11651	212203_x_at	interferon induced transmembrane protein 3 (1-8U)
MGC16202	2,5	0,000	26196	226326_at	hypothetical protein MGC16202
LST1	2,5	0,000	14014	214574_x_at	leukocyte specific transcript 1
AIG1	2,5	0,000	23028	223136_at	androgen-induced 1
C3AR1	2,4	0,019	9454	209906_at	complement component 3a receptor 1
FLJ00332	2,4	0,000	28126	228258_at	FLJ00332 protein
LOC122618	2,4	0,000	35665	235802_at	hypothetical protein BC015003

CTSC	2,4	0,000	25517	225646_at	cathepsin C
GBP4	2,4	0,049	35038	235175_at	guanylate binding protein 4
CX3CR1	2,4	0,041	5487	205898_at	chemokine (C-X3-C motif) receptor 1
LILRB1	2,4	0,000	29805	229937_x_at	leukocyte immunoglobulin-like receptor, subfamily B (with TM and ITIM domains), member 1
ITGAL	2,4	0,000	12918	213475_s_at	integrin, alpha L (antigen CD11A (p180)
PSCDBP	2,3	0,000	9158	209606_at	pleckstrin homology, Sec7 and coiled-coil domains, binding protein
SLAMF7	2,3	0,000	22731	222838_at	SLAM family member 7
KIAA1039	2,3	0,000	12723	213280_at	KIAA1039 protein
CDC42EP4	2,3	0,000	14159	214721_x_at	CDC42 effector protein (Rho GTPase binding) 4
LYN	2,3	0,000	2216	202626_s_at	v-yes-1 Yamaguchi sarcoma viral related oncogene homolog
HSPH1	2,3	0,048	6564	206976_s_at	heat shock 105kDa/110kDa protein 1
GNG2	2,3	0,000	24836	224964_s_at	guanine nucleotide binding protein (G protein), gamma 2
HT021	2,3	0,000	18714	219288_at	HT021
MAP4	2,3	0,000	21806	243_g_at	microtubule-associated protein 4
C6orf187	2,3	0,022	29258	229390_at	chromosome 6 open reading frame 187
ULK2	2,3	0,000	3651	204062_s_at	unc-51-like kinase 2 (C. elegans)
ABHD6	2,3	0,000	22074	45288_at	abhydrolase domain containing 6
HHL	2,3	0,000	14778	215342_s_at	expressed in hematopoietic cells, heart, liver
ETS1	2,3	0,000	24705	224833_at	v-ets erythroblastosis virus E26 oncogene homolog 1 (avian)
CXCL16	2,3	0,000	23345	223454_at	chemokine (C-X-C motif) ligand 16
KLF2	2,3	0,000	18797	219371_s_at	Kruppel-like factor 2 (lung)
DUSP7	2,3	0,000	13290	213848_at	dual specificity phosphatase 7
WARS	2,3	0,000	219	200629_at	tryptophanyl-tRNA synthetase
CD47	2,3	0,000	25886	226016_at	CD47 antigen (Rh-related antigen, integrin-associated signal transducer)
RARA	2,3	0,000	3338	203749_s_at	retinoic acid receptor, alpha
UNC93B1	2,3	0,000	20424	220998_s_at	unc-93 homolog B1 (C. elegans) /// unc-93 homolog B1 (C. elegans)
RASGRP2	2,3	0,000	7773	208206_s_at	RAS guanyl releasing protein 2 (calcium and DAG-regulated)
GCH1	2,2	0,022	3813	204224_s_at	GTP cyclohydrolase 1 (dopa-responsive dystonia)
MADH1	2,2	0,000	27666	227798_at	MAD, mothers against decapentaplegic homolog 1 (Drosophila)
REPS1	2,2	0,000	24246	224366_s_at	RALBP1 associated Eps domain containing 1
AP2A1	2,2	0,000	33932	234068_s_at	adaptor-related protein complex 2, alpha 1 subunit
IL12RB1	2,2	0,000	44992	1552584_at	interleukin 12 receptor, beta 1
LFNG	2,2	0,000	28630	228762_at	lunatic fringe homolog (Drosophila)
SWAP70	2,2	0,000	8863	209307_at	SWAP-70 protein
DRAP1	2,2	0,000	2847	203258_at	DR1-associated protein 1 (negative cofactor 2 alpha)
WDR11	2,2	0,000	17517	218090_s_at	WD repeat domain 11
ARRB1	2,2	0,000	18258	218832_x_at	arrestin, beta 1
SLC1A4	2,2	0,000	12258	212811_x_at	solute carrier family 1 (glutamate/neutral amino acid transporter), member 4
CD97	2,2	0,000	2501	202910_s_at	CD97 antigen
GRCC10	2,2	0,000	24592	224719_s_at	likely ortholog of mouse gene rich cluster, C10 gene
RAFTLIN	2,2	0,000	12093	212646_at	raft-linking protein
SVIL	2,2	0,000	2155	202565_s_at	supervillin

MGLL	2,2	0,000	10534	211026_s_at	monoglyceride lipase /// monoglyceride lipase
ARHF	2,2	0,000	18471	219045_at	ras homolog gene family, member F (in filopodia)
NELF	2,2	0,000	20639	221214_s_at	nasal embryonic luteinizing hormone-releasing hormone factor
PIK3AP1	2,1	0,000	26329	226459_at	phosphoinositide-3-kinase adaptor protein 1
MAIL	2,1	0,000	23109	223217_s_at	molecule possessing ankyrin repeats induced by lipopolysaccharide (MAIL), homolog of mouse
CKS1B	2,1	0,000	1487	201897_s_at	CDC28 protein kinase regulatory subunit 1B
CENTD1	2,1	0,000	13060	213618_at	centaurin, delta 1
POU2F2	2,1	0,041	28211	228343_at	POU domain, class 2, transcription factor 2
TRIM14	2,1	0,022	2738	203148_s_at	tripartite motif-containing 14
VIL2	2,1	0,022	8182	208623_s_at	villin 2 (ezrin)
PECAM1	2,1	0,000	8539	208983_s_at	platelet/endothelial cell adhesion molecule (CD31 antigen)
BCL2A1	2,1	0,022	5270	205681_at	BCL2-related protein A1
NFATC1	2,1	0,000	9708	210162_s_at	nuclear factor of activated T-cells, cytoplasmic, calcineurin-dependent 1
RAPH1	2,1	0,029	25059	225188_at	Ras association (RalGDS/AF-6) and pleckstrin homology domains 1
MCOLN1	2,1	0,000	19378	219952_s_at	mucolipin 1
PEX7	2,1	0,000	5009	205420_at	peroxisomal biogenesis factor 7
LIM	2,1	0,000	16236	216804_s_at	LIM protein (similar to rat protein kinase C-binding enigma)
P114-RHO-GEF	2,1	0,000	12484	213039_at	Rho-specific guanine nucleotide exchange factor p114
FLJ11029	2,1	0,000	28141	228273_at	hypothetical protein FLJ11029
RYBP	2,1	0,022	1435	201845_s_at	RING1 and YY1 binding protein
FLJ21069	2,1	0,000	26295	226425_at	hypothetical protein FLJ21069
VDR	2,0	0,000	3844	204255_s_at	vitamin D (1,25- dihydroxyvitamin D3) receptor
NEURL	2,0	0,000	4477	204888_s_at	neuralized-like (Drosophila)
ZBTB4	2,0	0,000	25500	225629_s_at	zinc finger and BTB domain containing 4
UBE2J1	2,0	0,000	17251	217824_at	ubiquitin-conjugating enzyme E2, J1 (UBC6 homolog, yeast)
GPR155	2,0	0,000	31034	231166_at	G protein-coupled receptor 154
SH3MD2	2,0	0,000	25460	225589_at	SH3 multiple domains 2
LOC339903	2,0	0,038	28589	228721_at	hypothetical protein LOC339903
TMPO	2,0	0,022	24816	224944_at	thymopoietin
ZRANB1	2,0	0,022	41999	242136_x_at	zinc finger, RAN-binding domain containing 1
NESH	2,0	0,000	23505	223615_at	NESH protein

Unknown transcribed sequences (n=19)

GeneSymbol	ratio	p-value	ProbeSetN	ProbeSetID	GeneTitle
---	7,4	0,000	16326	216894_x_at	---
---	5,4	0,000	15944	216511_s_at	---
---	3,1	0,022	15997	216565_x_at	---
---	3,0	0,000	39157	239294_at	Homo sapiens transcribed sequences
---	2,8	0,000	48998	1558397_at	Homo sapiens cDNA FLJ34100 fis, clone FCBBF3007597.
---	2,7	0,000	13167	213725_x_at	Homo sapiens, clone IMAGE:4791553, mRNA
---	2,6	0,000	27730	227862_at	Homo sapiens LOC374963 (LOC374963), mRNA

---	2,5	0,000	43810	243947_s_at	Homo sapiens transcribed sequences
---	2,5	0,000	24973	225102_at	Homo sapiens cDNA clone IMAGE:4441572, partial cds
---	2,3	0,000	29374	229506_at	Homo sapiens cDNA FLJ30761 fis, clone FEBRA2000538.
---	2,3	0,000	27159	227290_at	Homo sapiens cDNA FLJ13598 fis, clone PLACE1009921.
---	2,2	0,000	28312	228444_at	Homo sapiens mRNA; cDNA DKFZp762M127 (from clone DKFZp762M127)
---	2,2	0,049	32049	232181_at	Homo sapiens mRNA; cDNA DKFZp686C1790 (from clone DKFZp686C1790)
---	2,1	0,000	27976	228108_at	Homo sapiens cDNA FLJ30761 fis, clone FEBRA2000538.
---	2,1	0,000	22805	222912_at	Homo sapiens mRNA; cDNA DKFZp762M127 (from clone DKFZp762M127)
---	2,1	0,000	49499	1559263_s_at	Homo sapiens similar to hypothetical protein D730019B10 (LOC340152), mRNA
---	2,1	0,000	48005	1556747_a_at	Homo sapiens cDNA FLJ39784 fis, clone SPLEN2002314.
---	2,0	0,039	52748	1565743_at	Homo sapiens, clone IMAGE:4418644, mRNA
---	2,0	0,048	36061	236198_at	Homo sapiens transcribed sequences
