# Supplementary material for: Transcriptional profiling reveals developmental relationship and distinct biological functions of CD16+ and CD16- monocyte subsets
Source: BMC Genomics. 2009 Aug 27;10:403. doi: 10.1186/1471-2164-10-403 (PMC2741492; doi:10.1186/1471-2164-10-403)
Supplement: Additional file 2 — Table S2. Genes downregulated in CD16+ compared to CD16- monocytes. Calculation of expression ratios for the 2,759 differentially expressed probe sets showed downregulation of 250 probe sets (corresponding to 166 genes and 23 unknown transcribed sequences) in CD16+ compared to CD16- Mo (cut-off 2-fold; p < 0.05). [file 1471-2164-10-403-S2.pdf]

Supplemental Table 2 - Genes downregulated in CD16+ monocytes

Known genes (n=166)

GeneSymbol	ratio	p-value	ProbeSetN	ProbeSetID	GeneTitle
S100A12	0,12	0,000	5452	205863_at	S100 calcium binding protein A12 (calgranulin C)
CSPG2	0,13	0,000	11038	211571_s_at	chondroitin sulfate proteoglycan 2 (versican)
CD163	0,17	0,000	3234	203645_s_at	CD163 antigen
MGC71745	0,17	0,000	26658	226789_at	similar to embigin
CD14	0,17	0,000	1333	201743_at	CD14 antigen
QPCT	0,18	0,000	4763	205174_s_at	glutaminyl-peptide cyclotransferase (glutaminyl cyclase)
LOC199675	0,18	0,000	35431	235568_at	hypothetical protein LOC199675
DKFZP434B044	0,19	0,000	20966	221541_at	hypothetical protein DKFZp434B044
SLC2A3	0,20	0,000	2089	202499_s_at	solute carrier family 2 (facilitated glucose transporter), member 3
CLECSF8	0,20	0,000	45141	1552773_at	C-type (calcium dependent, carbohydrate-recognition domain) lectin, superfamily member 8
MGST1	0,20	0,000	52606	1565162_s_at	microsomal glutathione S-transferase 1
CCR2	0,20	0,000	6566	206978_at	chemokine (C-C motif) receptor 2
RNASE2	0,21	0,000	5699	206111_at	ribonuclease, RNase A family, 2 (liver, eosinophil-derived neurotoxin)
PLA2G7	0,21	0,000	5802	206214_at	phospholipase A2, group VII (platelet-activating factor acetylhydrolase, plasma)
SLC2A14	0,21	0,000	21510	222088_s_at	solute carrier family 2 (facilitated glucose transporter), member 14
F13A1	0,21	0,000	2894	203305_at	coagulation factor XIII, A1 polypeptide
CD36	0,22	0,000	9109	209555_s_at	CD36 antigen (collagen type I receptor, thrombospondin receptor)
IER3	0,23	0,000	1221	201631_s_at	immediate early response 3
GPR27	0,23	0,000	27637	227769_at	G protein-coupled receptor 27
VNN2	0,24	0,000	5511	205922_at	vanin 2
THBS1	0,24	0,000	700	201110_s_at	thrombospondin 1
CD99	0,24	0,000	619	201029_s_at	CD99 antigen
CYP1B1	0,25	0,000	2027	202437_s_at	cytochrome P450, family 1, subfamily B, polypeptide 1
SLC40A1	0,25	0,000	22937	223044_at	solute carrier family 40 (iron-regulated transporter), member 1
FLJ31978	0,25	0,000	29638	229770_at	hypothetical protein FLJ31978
CLECSF9	0,25	0,000	22827	222934_s_at	C-type (calcium dependent, carbohydrate-recognition domain) lectin, superfamily member 9
IL13RA1	0,25	0,000	10413	210904_s_at	interleukin 13 receptor, alpha 1
ALDH1A1	0,25	0,000	11672	212224_at	aldehyde dehydrogenase 1 family, member A1
DREV1	0,26	0,000	17295	217868_s_at	DORA reverse strand protein 1
TREM1	0,26	0,000	18860	219434_at	triggering receptor expressed on myeloid cells 1
MS4A6A	0,26	0,000	24236	224356_x_at	membrane-spanning 4-domains, subfamily A, member 6A
CSF3R	0,26	0,000	45531	1553297_a_at	colony stimulating factor 3 receptor (granulocyte)
F5	0,27	0,000	4303	204714_s_at	coagulation factor V (proaccelerin, labile factor)
ACTN1	0,27	0,000	8195	208636_at	actinin, alpha 1
ALOX5AP	0,28	0,000	3763	204174_at	arachidonate 5-lipoxygenase-activating protein
CRTAP	0,28	0,000	46400	1554464_a_at	cartilage associated protein
GM2A	0,28	0,000	12184	212737_at	GM2 ganglioside activator protein
SMA3	0,29	0,000	6153	206565_x_at	SMA3

TFEC	0,29	0,000	6303	206715_at	transcription factor EC
FLJ22662	0,29	0,000	17881	218454_at	hypothetical protein FLJ22662
EREG	0,29	0,000	5356	205767_at	epiregulin
CAPG	0,30	0,000	1440	201850_at	capping protein (actin filament), gelsolin-like
BST1	0,30	0,000	5304	205715_at	bone marrow stromal cell antigen 1
FLJ21308	0,30	0,000	18459	219033_at	hypothetical protein FLJ21308
NCF1	0,30	0,000	4550	204961_s_at	neutrophil cytosolic factor 1 (47kDa, chronic granulomatous disease, autosomal 1)
MICAL2	0,30	0,000	11920	212473_s_at	flavoprotein oxidoreductase MICAL2
ITGAM	0,31	0,000	5375	205786_s_at	integrin, alpha M (complement component receptor 3, alpha; CD11b (p170)
PTPNS1	0,31	0,000	2487	202897_at	protein tyrosine phosphatase, non-receptor type substrate 1
SELL	0,31	0,038	4152	204563_at	selectin L (lymphocyte adhesion molecule 1)
IL6ST	0,32	0,000	11643	212195_at	interleukin 6 signal transducer (gp130, oncostatin M receptor)
HPSE	0,32	0,038	22774	222881_at	heparanase
RNASE6	0,33	0,000	13009	213566_at	ribonuclease, RNase A family, k6
FLJ10357	0,33	0,000	22191	58780_s_at	hypothetical protein FLJ10357
FLJ33069	0,34	0,000	38292	238429_at	hypothetical protein FLJ33069
C6orf192	0,34	0,000	26171	226301_at	chromosome 6 open reading frame 192
NRGN	0,34	0,000	3670	204081_at	neurogranin (protein kinase C substrate, RC3)
ID1	0,35	0,000	8494	208937_s_at	inhibitor of DNA binding 1, dominant negative helix-loop-helix protein
LMNB1	0,35	0,000	2865	203276_at	lamin B1
S100A9	0,36	0,000	3124	203535_at	S100 calcium binding protein A9 (calgranulin B)
CPNE2	0,36	0,000	25000	225129_at	copine II
IL1RN	0,36	0,000	12104	212657_s_at	interleukin 1 receptor antagonist
AHR	0,36	0,000	2410	202820_at	aryl hydrocarbon receptor
GPX1	0,36	0,000	326	200736_s_at	glutathione peroxidase 1
FBN2	0,37	0,000	2774	203184_at	fibrillin 2 (congenital contractural arachnodactyly)
PLP2	0,37	0,000	726	201136_at	proteolipid protein 2 (colonic epithelium-enriched)
CD1D	0,37	0,000	5378	205789_at	CD1D antigen, d polypeptide
KCTD12	0,37	0,000	11636	212188_at	potassium channel tetramerisation domain containing 12
NCF4	0,37	0,000	4736	205147_x_at	neutrophil cytosolic factor 4, 40kDa
TUBB1	0,37	0,025	30558	230690_at	tubulin, beta 1
C1QR1	0,38	0,000	2467	202877_s_at	complement component 1, q subcomponent, receptor 1
HOMER3	0,38	0,000	4236	204647_at	homer homolog 3 (Drosophila)
S100A8	0,38	0,000	2508	202917_s_at	S100 calcium binding protein A8 (calgranulin A)
KIAA1718	0,38	0,000	25013	225142_at	KIAA1718 protein
FLJ23153	0,38	0,000	25857	225987_at	likely ortholog of mouse tumor necrosis-alpha-induced adipose-related protein
VMP1	0,38	0,000	53742	1569003_at	likely ortholog of rat vacuole membrane protein 1
PPBP	0,38	0,025	13587	214146_s_at	pro-platelet basic protein (chemokine (C-X-C motif) ligand 7)
CPD	0,38	0,000	1530	201940_at	carboxypeptidase D
GPR160	0,38	0,000	23314	223423_at	G protein-coupled receptor 160
RAB27A	0,39	0,000	9070	209515_s_at	RAB27A, member RAS oncogene family
DKFZp762O076	0,39	0,000	26208	226338_at	hypothetical protein DKFZp762O076

FPR1	0,39	0,000	4708	205119_s_at	formyl peptide receptor 1
PADI4	0,39	0,026	19427	220001_at	peptidyl arginine deiminase, type IV
DSC2	0,39	0,000	26686	226817_at	desmocollin 2
PHLDA2	0,40	0,000	9352	209803_s_at	pleckstrin homology-like domain, family A, member 2
GRN	0,40	0,000	10775	211284_s_at	granulin
FLJ32798	0,40	0,000	38641	238778_at	hypothetical protein FLJ32798
EGFL5	0,40	0,000	12277	212830_at	EGF-like-domain, multiple 5
LOC91947	0,40	0,000	25154	225283_at	hypothetical protein LOC91947
FLJ23091	0,40	0,000	28817	228949_at	putative NFkB activating protein 373
FCGR1A	0,40	0,000	16382	216950_s_at	Fc fragment of IgG, high affinity Ia, receptor for (CD64)
JAG1	0,40	0,000	8655	209099_x_at	jagged 1 (Alagille syndrome)
IL1B	0,41	0,029	4656	205067_at	interleukin 1, beta
HEXB	0,41	0,000	1534	201944_at	hexosaminidase B (beta polypeptide)
FLJ10359	0,41	0,000	8490	208933_s_at	hypothetical protein FLJ10359
MNDA	0,41	0,000	4548	204959_at	myeloid cell nuclear differentiation antigen
PTAFR	0,41	0,000	5866	206278_at	platelet-activating factor receptor
APLP2	0,41	0,000	14312	214875_x_at	amyloid beta (A4) precursor-like protein 2
SGK	0,41	0,000	1329	201739_at	serum/glucocorticoid regulated kinase
MGC39820	0,41	0,000	25810	225940_at	hypothetical protein MGC39820
HSPC195	0,42	0,000	24391	224516_s_at	hypothetical protein HSPC195 /// hypothetical protein HSPC195
KIAA0146	0,42	0,025	28193	228325_at	KIAA0146 protein
STAB1	0,42	0,000	21979	38487_at	stabilin 1
PRKAR2B	0,42	0,000	3269	203680_at	protein kinase, cAMP-dependent, regulatory, type II, beta
C1orf24	0,42	0,038	17394	217967_s_at	chromosome 1 open reading frame 24
C9orf19	0,43	0,000	25475	225604_s_at	chromosome 9 open reading frame 19
IRF2BP2	0,43	0,000	24445	224572_s_at	interferon regulatory factor 2 binding protein 2
DKFZp434L142	0,43	0,000	19298	219872_at	hypothetical protein DKFZp434L142
BLVRB	0,43	0,000	1791	202201_at	biliverdin reductase B (flavin reductase (NADPH))
GCA	0,43	0,000	3354	203765_at	grancalcin, EF-hand calcium binding protein
TPCN1	0,43	0,000	17341	217914_at	two pore segment channel 1
PLSCR1	0,43	0,000	41779	241916_at	phospholipid scramblase 1
MAFF	0,44	0,000	21913	36711_at	v-maf musculoaponeurotic fibrosarcoma oncogene homolog F (avian)
TNFSF8	0,44	0,000	35598	235735_at	tumor necrosis factor (ligand) superfamily, member 8
MGC26963	0,44	0,038	26907	227038_at	hypothetical protein MGC26963
CIDEB	0,44	0,000	20613	221188_s_at	cell death-inducing DFFA-like effector b
APP	0,44	0,000	14390	214953_s_at	amyloid beta (A4) precursor protein (protease nexin-II, Alzheimer disease)
CKAP4	0,44	0,000	589	200999_s_at	cytoskeleton-associated protein 4
PSTPIP1	0,45	0,000	10682	211178_s_at	proline-serine-threonine phosphatase interacting protein 1
FAM13A1	0,45	0,000	16479	217047_s_at	family with sequence similarity 13, member A1
C6orf56	0,45	0,000	42140	242277_at	chromosome 6 open reading frame 56
GRSP1	0,45	0,000	12501	213056_at	GRP1-binding protein GRSP1
USP15	0,45	0,000	10207	210681_s_at	ubiquitin specific protease 15

TM6SF1	0,45	0,025	19318	219892_at	transmembrane 6 superfamily member 1
PTPNS1L3	0,45	0,027	49367	1559034_at	protein tyrosine phosphatase, non-receptor type substrate 1-like 3
SLC38A2	0,45	0,000	22875	222982_x_at	solute carrier family 38, member 2
SAP30	0,45	0,000	4489	204900_x_at	sin3-associated polypeptide, 30kDa
NFE2	0,45	0,000	9478	209930_s_at	nuclear factor (erythroid-derived 2), 45kDa
GRN	0,45	0,000	15475	216041_x_at	granulin
TNFAIP3	0,46	0,000	2234	202644_s_at	tumor necrosis factor, alpha-induced protein 3
FLJ20186	0,46	0,000	25508	225637_at	hypothetical protein FLJ20186
IL6R	0,46	0,000	26203	226333_at	interleukin 6 receptor
MAP2K6	0,46	0,000	5287	205698_s_at	mitogen-activated protein kinase kinase 6
PPIF	0,46	0,000	1079	201489_at	peptidylprolyl isomerase F (cyclophilin F)
LYZ	0,46	0,038	46379	1555745_a_at	lysozyme (renal amyloidosis)
MAML2	0,46	0,038	35320	235457_at	mastermind-like 2 (Drosophila)
MYCL1	0,46	0,000	13499	214058_at	v-myc myelocytomatosis viral oncogene homolog 1, lung carcinoma derived (avian)
AQP9	0,46	0,000	5157	205568_at	aquaporin 9
PGD	0,46	0,000	708	201118_at	phosphogluconate dehydrogenase /// phosphogluconate dehydrogenase
SORL1	0,46	0,000	3098	203509_at	sortilin-related receptor, L(DLR class) A repeats-containing
KIAA1598	0,46	0,000	21224	221802_s_at	KIAA1598 protein
LENG4	0,47	0,000	8735	209179_s_at	leukocyte receptor cluster (LRC) member 4
RIT1	0,47	0,000	36087	236224_at	Ras-like without CAAX 1
ALDH2	0,47	0,000	1015	201425_at	aldehyde dehydrogenase 2 family (mitochondrial)
P4HB	0,47	0,000	52433	1564494_s_at	procollagen-proline, 2-oxoglutarate 4-dioxygenase (proline 4-hydroxylase), beta polypeptide
SMA5	0,47	0,000	14479	215043_s_at	SMA5
IDH1	0,47	0,000	783	201193_at	isocitrate dehydrogenase 1 (NADP+), soluble
OGFRL1	0,47	0,000	38332	238469_at	opioid growth factor receptor-like 1
CCR1	0,47	0,032	4688	205099_s_at	chemokine (C-C motif) receptor 1
HIF1A	0,47	0,000	579	200989_at	hypoxia-inducible factor 1, alpha subunit (basic helix-loop-helix transcription factor)
MSCP	0,48	0,000	26049	226179_at	mitochondrial solute carrier protein
CGI-07	0,48	0,000	17463	218036_x_at	CGI-07 protein
TALDO1	0,48	0,000	1053	201463_s_at	transaldolase 1
TGIF	0,48	0,000	2902	203313_s_at	TGFB-induced factor (TALE family homeobox)
BCL3	0,48	0,000	4497	204908_s_at	B-cell CLL/lymphoma 3
SGSH	0,48	0,000	21891	35626_at	N-sulfoglucosamine sulfohydrolase (sulfamidase)
LOC340061	0,48	0,000	24788	224916_at	hypothetical protein LOC340061
ADM	0,48	0,000	2503	202912_at	adrenomedullin
FLJ21868	0,48	0,000	18074	218648_at	hypothetical protein FLJ21868
EVI2A	0,49	0,000	4363	204774_at	ecotropic viral integration site 2A
BNIP3L	0,49	0,000	20903	221478_at	BCL2/adenovirus E1B 19kDa interacting protein 3-like
AMICA	0,49	0,000	27962	228094_at	adhesion molecule AMICA
LOC285550	0,50	0,000	27335	227466_at	hypothetical protein LOC285550
MOB4A	0,50	0,000	25867	225997_at	Mob4A protein
WSX1	0,50	0,000	21484	222062_at	class I cytokine receptor

DPYD	0,50	0,000	4235	204646_at	dihydropyrimidine dehydrogenase
PPGB	0,50	0,000	251	200661_at	protective protein for beta-galactosidase (galactosialidosis)

Unknown transcribed sequences (n=23)

GeneSymbol	ratio	p-value	ProbeSetN	ProbeSetID	GeneTitle
---	0,50	0,000	29802	229934_at	Homo sapiens transcribed sequences
---	0,49	0,000	14939	215504_x_at	Homo sapiens clone 25061 mRNA sequence
---	0,48	0,027	39374	239511_s_at	Homo sapiens transcribed sequence with weak similarity to protein sp:P39194 (H.sapiens)
---	0,48	0,000	38952	239089_at	Homo sapiens transcribed sequences
---	0,47	0,000	26300	226430_at	Homo sapiens cDNA: FLJ21778 fis, clone HEP00201
---	0,47	0,000	53521	1568609_s_at	Homo sapiens, clone IMAGE:6061696, mRNA
---	0,47	0,000	41301	241438_at	Homo sapiens transcribed sequences
---	0,45	0,000	38376	238513_at	---
---	0,44	0,000	14507	215071_s_at	---
---	0,44	0,000	13372	213931_at	Homo sapiens transcribed sequence with strong similarity to protein pir:A40227 (H.sapiens)
---	0,44	0,027	34891	235028_at	Homo sapiens cDNA FLJ46440 fis, clone THYMU3016022
---	0,42	0,000	49871	1559910_at	Homo sapiens mRNA full length insert cDNA clone EUROIMAGE 151282
---	0,42	0,000	29096	229228_at	Homo sapiens cDNA FLJ32589 fis, clone SPLEN2000443.
---	0,42	0,000	12259	212812_at	Homo sapiens cDNA: FLJ22642 fis, clone HSI06970
---	0,41	0,000	34935	235072_s_at	Homo sapiens transcribed sequences
---	0,41	0,000	28244	228376_at	Homo sapiens, clone IMAGE:4812754, mRNA
---	0,40	0,000	48825	1558011_at	Homo sapiens transcribed sequence with moderate similarity to protein ref:NP_055163.1 (H.sapiens)
---	0,39	0,000	35661	235798_at	Homo sapiens transcribed sequence with weak similarity to protein ref:NP_055301.1 (H.sapiens)
---	0,37	0,000	32099	232231_at	Homo sapiens mRNA; cDNA DKFZp761J1112 (from clone DKFZp761J1112)
---	0,34	0,000	49952	1560034_a_at	Homo sapiens mRNA; cDNA DKFZp313E1515 (from clone DKFZp313E1515)
---	0,33	0,000	11079	211612_s_at	---
---	0,32	0,000	4449	204860_s_at	Homo sapiens transcribed sequence with strong similarity to protein sp:Q13075 (H.sapiens)
---	0,27	0,000	13525	214084_x_at	Homo sapiens similar to Neutrophil cytosolic factor 1 (LOC378112), mRNA
