# Supplementary figures and images for: Transcriptional profiling reveals developmental relationship and distinct biological functions of CD16+ and CD16- monocyte subsets
Source: BMC Genomics. 2009 Aug 27;10:403. doi: 10.1186/1471-2164-10-403 (PMC2741492; doi:10.1186/1471-2164-10-403)

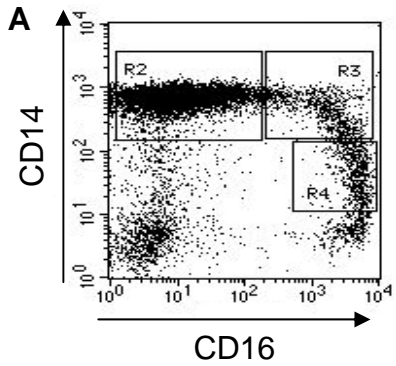

— Control  
 — CD14<sup>high</sup>CD16<sup>neg</sup> Mo (R2)  
 — CD14<sup>high</sup>CD16<sup>+</sup> Mo (R3)  
 — CD14<sup>low</sup>CD16<sup>+</sup> Mo (R4)

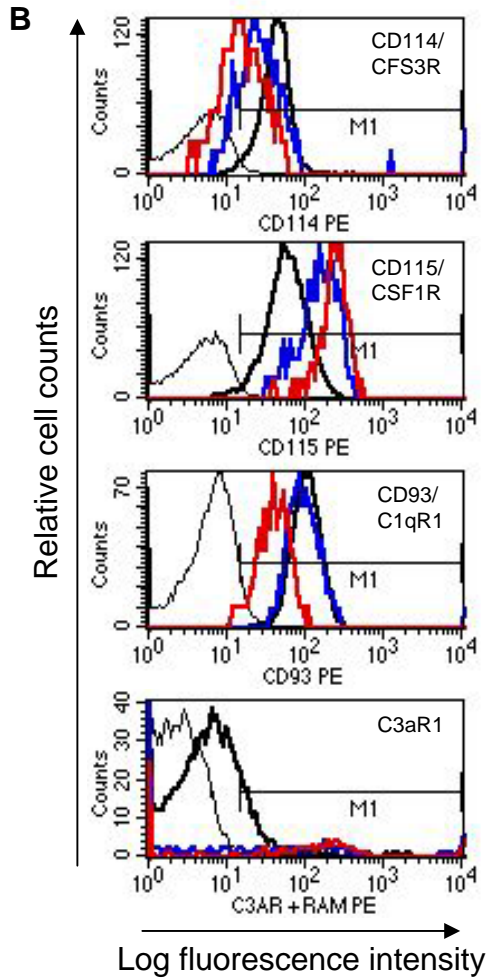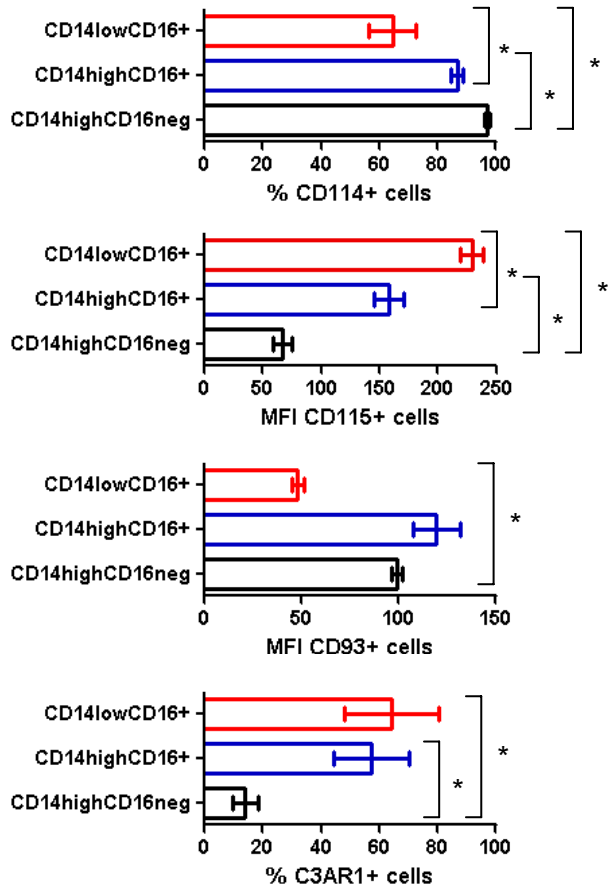

Supplement: Additional file 3 — Figure S1. Differential expression of CD114/CSF3R, CD115/CSF1R, CD93/C1qR1 and C3aR1 on CD14highCD16-, CD14highCD16+, and CD14lowCD16+ monocytes. Freshly isolated PBMC were stained with FITC CD14, PE-Cy5 CD16, and PE CD114, PE CD115, and PE CD93 Abs. The expression of CD3aR1 was detected after staining with unconjugated mouse C3AR1 Ab and PE rat anti-mouse Ab (RAM). CD14highCD16neg (R2), CD14highCD16+ (R3) and CD14lowCD16+ (R4) Mo (A) were analyzed for expression of CD114, CD115, CD93 and C3aR1 (B). Shown is an overlay histogram from one representative donor of 4 donors examined (B, left panels) and graphs showing mean ± SEM for % or MFI of CD114, CD115, CD93, and C3aR1 expression on each Mo subset (B, right panels). (*, Paired t-test p-values < 0.05, CD16+ versus CD16- Mo; n = 4). [file 1471-2164-10-403-S3.pdf]
